# Nrl Is Dispensable for Specification of Rod Photoreceptors in Adult Zebrafish Despite Its Deeply Conserved Requirement Earlier in Ontogeny

**DOI:** 10.1016/j.isci.2020.101805

**Published:** 2020-11-15

**Authors:** A. Phillip Oel, Gavin J. Neil, Emily M. Dong, Spencer D. Balay, Keon Collett, W. Ted Allison

**Affiliations:** 1 Department of Biological Sciences, University of Alberta, Edmonton AB, T7Y 1C4, Canada; 2 Department of Medical Genetics, University of Alberta, Edmonton AB, T6G 2R3, Canada

**Keywords:** Biological Sciences, Molecular Biology, Developmental Biology

## Abstract

The transcription factor NRL (neural retina leucine zipper) has been canonized as the master regulator of photoreceptor cell fate in the retina. NRL is necessary and sufficient to specify rod cell fate and to preclude cone cell fate in mice. By engineering zebrafish, we tested if NRL function has conserved roles beyond mammals or beyond nocturnal species, i.e., in a vertebrate possessing a greater and more typical diversity of cone sub-types. Transgenic expression of Nrl from zebrafish or mouse was sufficient to induce rod photoreceptor cells. Zebrafish *nrl*^*−/−*^ mutants lacked rods (and had excess UV-sensitive cones) as young larvae; thus, the conservation of Nrl function between mice and zebrafish appears sound. Strikingly, however, rods were abundant in adult *nrl*^*−/−*^ null mutant zebrafish. Rods developed in adults despite Nrl protein being undetectable. Therefore, a yet-to-be-revealed non-canonical pathway independent of Nrl is able to specify the fate of some rod photoreceptors.

## Highlights

• Nrl is conserved and sufficient to specify rod photoreceptors in the zebrafish retina
• Nrl is necessary for rod photoreceptors in early ontogeny of zebrafish larvae
• Zebrafish Nrl is functionally conserved with mouse and human NRL
• Remarkably, Nrl is dispensable for rod specification in adult zebrafish

## Graphical Abstract

**Figure:**
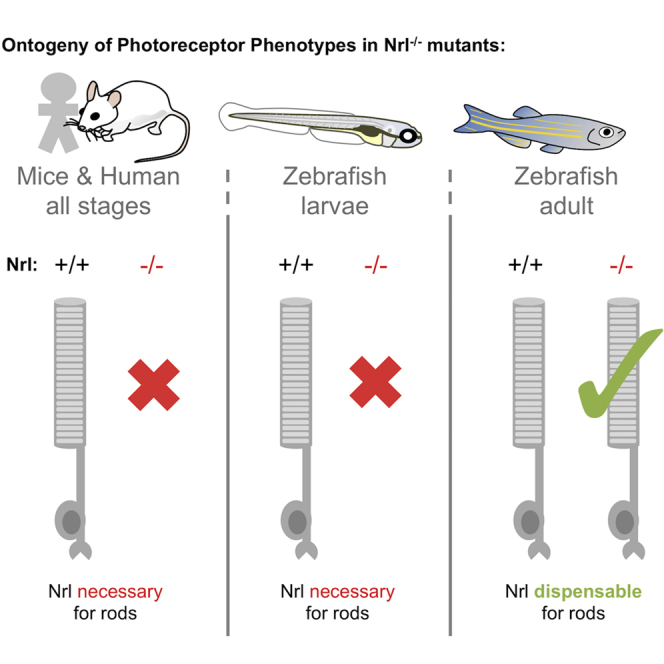

## Introduction

Rods and cones are the ciliary photoreceptors used by vertebrates to enable vision across a broad range of circumstances. Rod photoreceptors enable vision in dim conditions, while cone photoreceptors convey wavelength-specific information, enable high acuity, and can operate in brightly lit environments. Retinas with both rods and cones are known as duplex retinas, and the basic features of the duplex retina are present even among some of the earliest branching vertebrates, the lampreys (Asteriti et al., 2015; Collin and Trezise, 2004; Morshedian and Fain, 2015).

**Figure 1:**
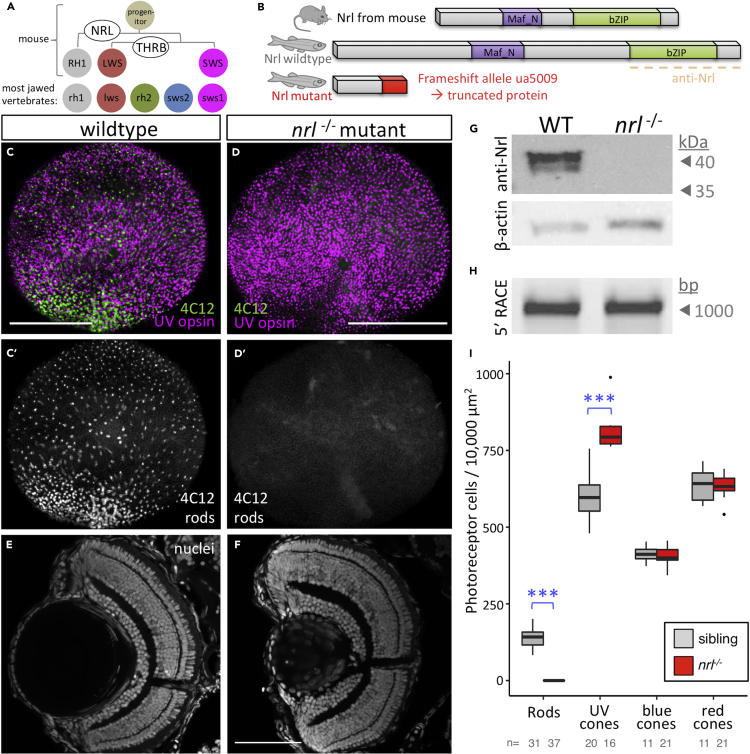
Nrl Is Conserved and Required for Rod Specification in Larval Zebrafish (A) Nrl is the master regulator of photoreceptor specification in mice, being both necessary and sufficient for rod photoreceptor development from progenitor cells. Elegantly simple models can account for cell fate specification and generating the full complement of photoreceptor types in mice (and all mammals studied) using only two factors, NRL and THRB (thyroid hormone receptor β), to generate rods, red cones, and blue cones expressing RH1, LWS, and SWS opsins, respectively. However, most vertebrates possess additional cone subtypes. (B) Zebrafish Nrl protein is recognizably similar to mammalian homologs. An *nrl*^−/−^ null mutation was engineered via CRISPR that is predicted to truncate the protein (see also Figure S1). (C and D) CRISPR-engineered null Nrl mutants lack rods in larval zebrafish, matching the phenotype of adult *NRL*^*−/−*^ knockout mice. Flat-mounted retina from larval zebrafish show that rods are absent in *nrl*^−/−^ larvae, as detected by rod immunomarker 4C12. The typical broad distribution of rods (B′) is absent (C′) and the decrease in rod cell abundance is consistent, nearly complete and robustly significant (I). (E and F) Retina of *nrl*^−/−^ larvae is broadly normal in its lamination. (G) Nrl protein is not detectable in *nrl*^−/−^ retina, and antibody validation is supported by a doublet band (presumably reflecting SUMOylation as per mammalian homologs of Nrl) appearing at the predicted size of 44.2 kDa (See Figure S1 for full immunoblots and quantification). (H) Assessment of *nrl* transcript by 5′RACE (random amplification of cDNA ends) does not reveal any unexpected transcripts such as those with cryptic exons that could be imagined to make functional protein. (I) Quantification of photoreceptor types in *nrl*^−/−^ larvae confirms the consistent absence of rod cells and a concomitant increase in UV cone abundance. Box and whisker plots first through third quartile and distribution of data, respectively, after excluding outliers. ∗∗∗p < 0.001; bar in D is 100 um. The number of individual larvae examined (n) is indicated.

The visual photoreceptors are among the best-studied neurons with respect to developmental programs and gene regulatory networks. Photoreceptor precursor cells of the developing mouse retina are thoroughly studied, and an elegantly simple gene regulatory network determines all rod and cone cell fates. As the precursor cell exits its terminal mitosis, expression of neural retina leucine zipper (NRL) (a basic leucine zipper transcription factor) directs the cell to a rod fate (schematized in Figure 1A); without NRL expression, it develops as a cone (Mears et al., 2001; Daniele et al., 2005; Nikonov et al., 2005; Swaroop et al., 2010). With high activity of the thyroid hormone receptor β (THRB), the presumptive cone will develop into the medium (green) wavelength light-sensitive M-cone (the ancestral red cone, expressing long-wavelength-sensitive (LWS) opsin). Without THRB activity, it becomes a short wavelength (UV/blue) light-sensitive S-cone (the ancestral UV cone expressing SWS1 opsin) (Ng et al., 2001). This efficient two-factor specification model is expected to be sufficient to generate all photoreceptor diversity in most all eutherian mammals, which have lost the ancestral blue and green light-sensitive cone subtypes (Ng et al., 2011). In typical non-mammalian vertebrates that have four cone subtypes (and rods), the blue and green cone specification remains unexplained. Thyroid hormone and likely its receptor TRB2 control red cone development in chicken (Vancamp et al., 2019), and *trb2* regulates red cone versus UV cone fate in zebrafish (DuVal and Allison, 2018; Yoshimatsu et al., 2014; Mackin et al., 2019), demonstrating substantial conservation of the photoreceptor specification program in tetrachromats. However, homologs of NRL have not been disrupted or manipulated sufficiently to appreciate its role(s) in species outside of mammals.

Previous work has shown that *nrl* is expressed in or near the rod photoreceptors of zebrafish larvae (Coolen et al., 2005; Nelson et al., 2008). Xenopus embryos expressing lipofected Xenopus *Nrl* showed rhodopsin immunoreactivity in lipofected cells (McIlvain and Knox, 2007). This provides tentative support for *nrl* playing a role in photoreceptor development outside of mammals. However, while mouse *NRL* is expressed detectably in developing lens tissue (Liu et al., 1996), lipofected human *NRL* did not promote lens fiber cell differentiation as Xenopus *Nrl* did (McIlvain and Knox, 2007), suggesting divergent activities for the gene homologs. Underscoring this, the avian lineage has lost any detectable ortholog of *NRL* and likely relies on *MAFA* (a memebr of the *NRL* gene family, a long Maf)*MAFA* for rod specification (Ochi et al., 2004); *MAFA* is also expressed in non-photoreceptor retinal cells (Enright et al., 2015), as well as in lens tissues (Ochi et al., 2004), and can induce rods when ectopically expressed in mice (Kim et al., 2016).

We recently collaborated to compare lineage tracing events and prompt a new hypothesis that ancient mammals began to convert a large proportion of cone-fated progenitor cells to the rod cell fate (Kim et al., 2016). This was proposed as part of the mammalian adaptation to the nocturnal bottleneck (Kim et al., 2016), a phase of evolution in the earliest proto-mammals where they avoided daytime predators by adapting to a nocturnal lifestyle (Hall et al., 2012; Walls, 1942). We speculated that changes in NRL expression or function may have been involved in capturing cone cells to the rod fate. A burst of evolutionary change in NRL peptide sequence, well conserved among mammals but less so outside the clade (Kim et al., 2016), suggests a change in NRL functions and/or roles in development, as does the differing capacity for Xenopus *Nrl* versus human *NRL* to induce lens fiber differentiation in the frog (McIlvain and Knox, 2007).

To facilitate the comparison of photoreceptor specification programs between dichromat (mammalian) and tetrachromat (early-branching vertebrate) models, we challenged the hypothesis that the functional role of NRL is conserved between mouse and zebrafish. To this end, we determined the outcome of *nrl* loss on a tetrachromat retina across various stages of ontogeny. We also tested the capacity for ectopic zebrafish or mouse *NRL* orthologs to override an established cone-specified phenotype in favor of a rod phenotype in transgenic animals expressing *nrl* in UV cones. Moreover, we developed lineage tracing tools to track these outcomes over time. Overall, this paper identified both deeply conserved and diverging functional requirements for *NRL* between mice and zebrafish when considered over ontogeny.

## Results

### Larval Zebrafish Require *nrl* to Make Rods Early in Ontogeny

Zebrafish *nrl* is the sole zebrafish ortholog to mammalian *NRL*; no paralogs have been identified (Kim et al., 2016). The protein domains of zebrafish Nrl are conserved compared to mammals, though the zebrafish protein is longer in its primary sequence (Figures 1B and S1). We used CRISPR/Cas9-targeted mutagenesis to create a loss-of-function allele of zebrafish *nrl* and to assess its role in photoreceptor development (Figures 1B and S1). We generated frameshift allele *nrl*^*ua5009*^ that was predicted to lack all major Nrl protein domains and therefore was a putative null allele.

*nrl*^*ua5009*^ homozygous mutant zebrafish (referred to hereafter as *nrl*^*−/−*^) fail to produce rods at 4 days postfertilization (dpf) (Figures 1C and 1D), contrasting wild-type larvae that consistently produced a large abundance of rods by this time point. When examined using the rod-specific 4C12 antibody, we found that these *nrl* mutant zebrafish consistently contain zero rods within the entirety of the larval retina. *nrl*^*−/−*^ mutant zebrafish larval showed no overt abnormalities in retinal lamination (Figures 1E and 1F).

Immunoblots on wild-type zebrafish detected Nrl protein at the expected size (predicted to be 44.2 kDa based on primary sequence). Zebrafish Nrl immunoreactivity presented as a doublet band (Figure 1G) highly reminiscent of blots against mouse Nrl (Kim et al., 2012), where post-translational modification via SUMOylation has been determined experimentally to account for the doublet band (Roger et al., 2010). SUMOylation of zebrafish Nrl is plausible because the site of SUMOylation in human Nrl, residue K20, and surrounding residues (Roger et al., 2010) is exactly conserved in zebrafish (Figure S1A). Nrl protein was not detectable in *nrl*^*−/−*^ mutant retinas (Figure 1G and see Figure S1 for full blots and quantification). Consistent with the lack of detectable protein, 5′RACE (random amplification of cDNA ends) characterization showed no detectable alteration to splicing of the *nrl* transcript in *nrl*^*−/−*^ mutant larvae or adult retinas (Figure 1H and see Figure S1 for full blots). Thus, 5′RACE discounts possible confounds to the prediction of a null allele in our mutants (e.g. that might occur via imagined splicing of cryptic exons).

In mice, lack of *Nrl* causes overproduction of S-cone photoreceptors (in addition to loss of rod cells) (Mears et al., 2001). To assess if one or multiple of the zebrafish cone subtypes are more abundant in *nrl* mutants, we determined the relative abundance of photoreceptors of whole-mounted retinas. We found that rods were consistently absent in *nrl* mutants at 4 dpf (0 rods ±0 SEM, n = 37), whereas in wild-type larvae, within a 100 × 100μm region of interest, there were 141.45 rods ±5.76 (SEM, n = 29) (Figures 1D and 1I). We found that UV cones were significantly overproduced in mutants (mutant: 817.38 ± 26.60 [SEM]; wild-type: 599.64 ± 21.45 [SEM]; Mann-Whitney U test, p value = 0.0001512, U stat = 112) (Figures 1D and 1I). The excess UV cone abundance in *nrl* mutants was approximately equivalent to the normal abundance of rods in wild-type larvae, suggesting that cells otherwise fated to become rods might have become UV cones without *nrl*. Consistent with this, we did not detect a significant difference in blue cone or red cone abundance in *nrl* mutants (Figure 1I)

We did not detect overt morphological or developmental consequences of *nrl* mutation in zebrafish beyond the photoreceptor phenotypes documented above, aside from a disruption in the normal development of the lens. By 2 dpf, homozygous mutant zebrafish could be routinely distinguished from heterozygous or wild-type siblings by the presence of an occlusion in the lens (shown at 3 dpf, Figure S2). This is consistent with zebrafish *nrl* being robustly expressed in the developing lens fiber cells (Coolen et al., 2005).

**Figure 2:**
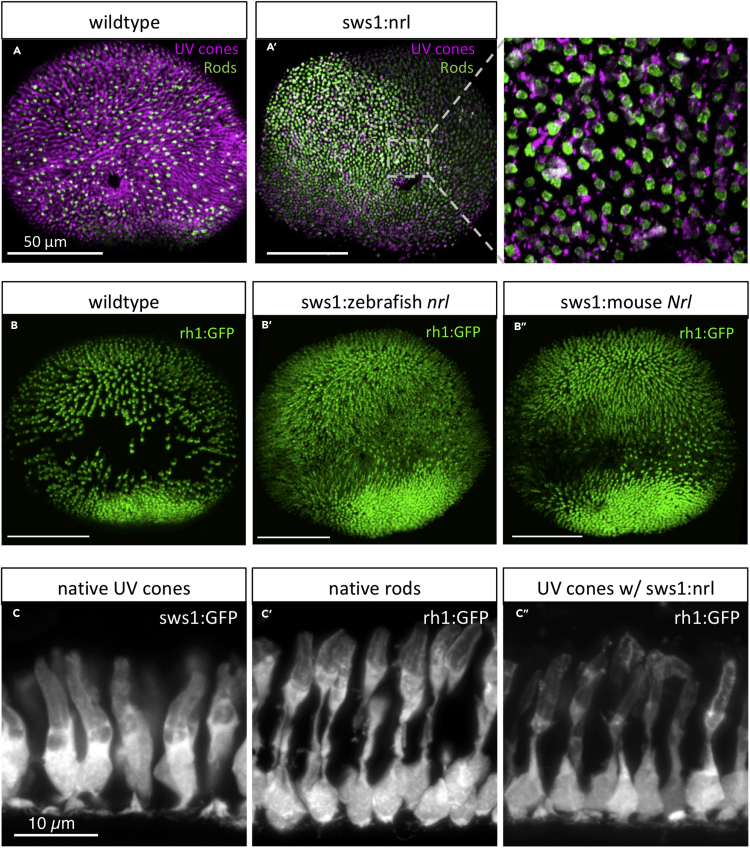
Zebrafish Nrl is Conserved and Sufficient to Induce Rod Photoreceptors in Zebrafish (A) Wild-type zebrafish larval retina in *en face* view has a dense forest of UV cone photoreceptor cells (magenta) and fewer rod cells (green) scattered throughout. (A’) Ectopic expression of zebrafish Nrl in differentiating UV cones transmutes these cells to a rod cell fate as determined by 4C12 immunoreactivity (green; 4 days postfertilization [dpf] larva). Inset: UV cones (magenta) are 4C12+ (green). (B) Rods are more abundant in wild-type zebrafish by 6dpf. (B”) Expression of mouse Nrl reroutes cones to a rod cell fate (defined as GFP + cells in *Tg[rh1:gfp]*) in a manner indistinguishable from zebrafish Nrl (B′). Note the increase in GFP-positive rods apparent in both lines (B′ & B″) relative to wild-type retina. (C) Cellular morphology of UV cones *vs.* rods (C *vs.* C′) is readily distinguishable by 7dpf in photoreceptor cells expressing GFP. (C″) Ectopic expression of zebrafish Nrl causes UV cones to take on a rod-like cell morphology. Larvae in (C″) are on a *nrl*^*−/−*^ null background (and thus lack native rod cells, described in Figure 3) to ensure the source of GFP-positive rod-like cells visualized here is the UV cones ectopically expressing the transgenic Nrl.

Controls for specificity of the above mutagenesis support that the frameshift lesion we induced in *nrl* is causal of the described photoreceptor phenotypes. Both phenotypes (reduction in rods, increase in UV cones) were recapitulated when *nrl* splicing was disrupted by morpholino (Figure S3). Furthermore, transgenic replacement of *nrl* was able to rescue the loss of rod cells on the *nrl*^*−/−*^ background (described immediately below and in Figure 2C″), arguing that our *nrl* mutant larvae possess all the molecular machinery required for producing rods (excepting *nrl* itself).

### Zebrafish *nrl* Is Conserved and Sufficient to Convert UV Cones to a Rod-like Fate

To test if zebrafish *nrl* is sufficient to induce a rod photoreceptor cell fate, we transgenically expressed *nrl* in developing zebrafish UV cones. We engineered transgenic zebrafish using regulatory sequences upstream of the *sws1* gene to drive expression of zebrafish *nrl*; this promoter has previously been characterized to drive expression exclusively in UV cones (Takechi et al., 2003; Kim et al., 2016; Fraser et al., 2013; Duval et al., 2014; Yoshimatsu et al., 2016; Suzuki et al., 2013). At 4 dpf, zebrafish with UV cones that expressed *nrl* showed 4C12 immunostaining (normally specific to rods) that colocalized with UV cones, as stained by anti-UV cone opsin (Figure 2A; exemplar image, the phenotype appears to be completely penetrant across dozens of larvae). Thus, zebrafish *nrl* is sufficient to produce rod photoreceptors.

Ectopic expression of zebrafish Nrl in differentiating cones was sufficient to induce the rod opsin promoter and rod cell fate, such that *Tg[rh1:GFP]* larvae (that express Green Fluorescent Protein [GFP], in rod cells) displayed a great density of GFP-positive cells (native rods and transmuted UV cones in Figure 2B. Images representative of dozens of larvae where the phenotype appears to be completely penetrant) compared to wild-type siblings (Figure 2B). Strikingly, the cellular morphology of the transmuted cones in *Tg[sws1:nrl]* retinas was nearly indistinguishable from native rods (Figure 2C″). Native rods can be readily distinguished from native cones (compare Figure 2C′ *vs*. 2C) by their long thin morphology connecting apical outer segments to the basal cell body and nuclear compartment (Figure 2C′), contrasting native UV cones that are more uniformly thick from their basal nucleus through their inner and outer segments (Figure 2C). To confirm that the *rh1:GFP*-positive rod-shaped cells characterized in Figure 2C″ were transmuted cones rather than native rods, these observations were made on an *nrl*^*−/−*^ background where native rods are absent (as described in Figure 1; the data are reminiscent of Nr2e3 rescuing rod cells in *Nrl*^*−/−*^ mice (Cheng et al., 2006); data from a single larva are presented). Overall then, diverse markers (Figures 2A, 2B, and 2C) show that zebrafish Nrl is sufficient to induce rod characters in differentiating zebrafish photoreceptors.

To further challenge the hypothesis that zebrafish Nrl is functionally conserved with mouse NRL, we examined retinas from larval fish engineered to be similar to those above (in Figure 2B′) but expressing the mouse Nrl gene in developing cones. Mouse Nrl converted UV cones to rods in a manner indistinguishable from zebrafish nrl, revealed by the high density of rods in transgenic *Tg[sws1:Mmu.NRL-FLAG]* retina compared to wild-type siblings (compare Figure 2B″ to B′ and their shared disparity compared to wild type in panel B). Together with previous data from transgenic mice, where ectopic expression of Nrl homologs from mouse or chicken were sufficient to induce rod cell fate (Oh et al., 2007; Kim et al., 2016), our data demonstrate that NRL homologs have a conserved capacity to induce rod cell fate across a diversity of vertebrates.

### Zebrafish Cone Cell Lineage has High Fidelity but can Be Induced to the Rod Cell Fate

**Figure 3:**
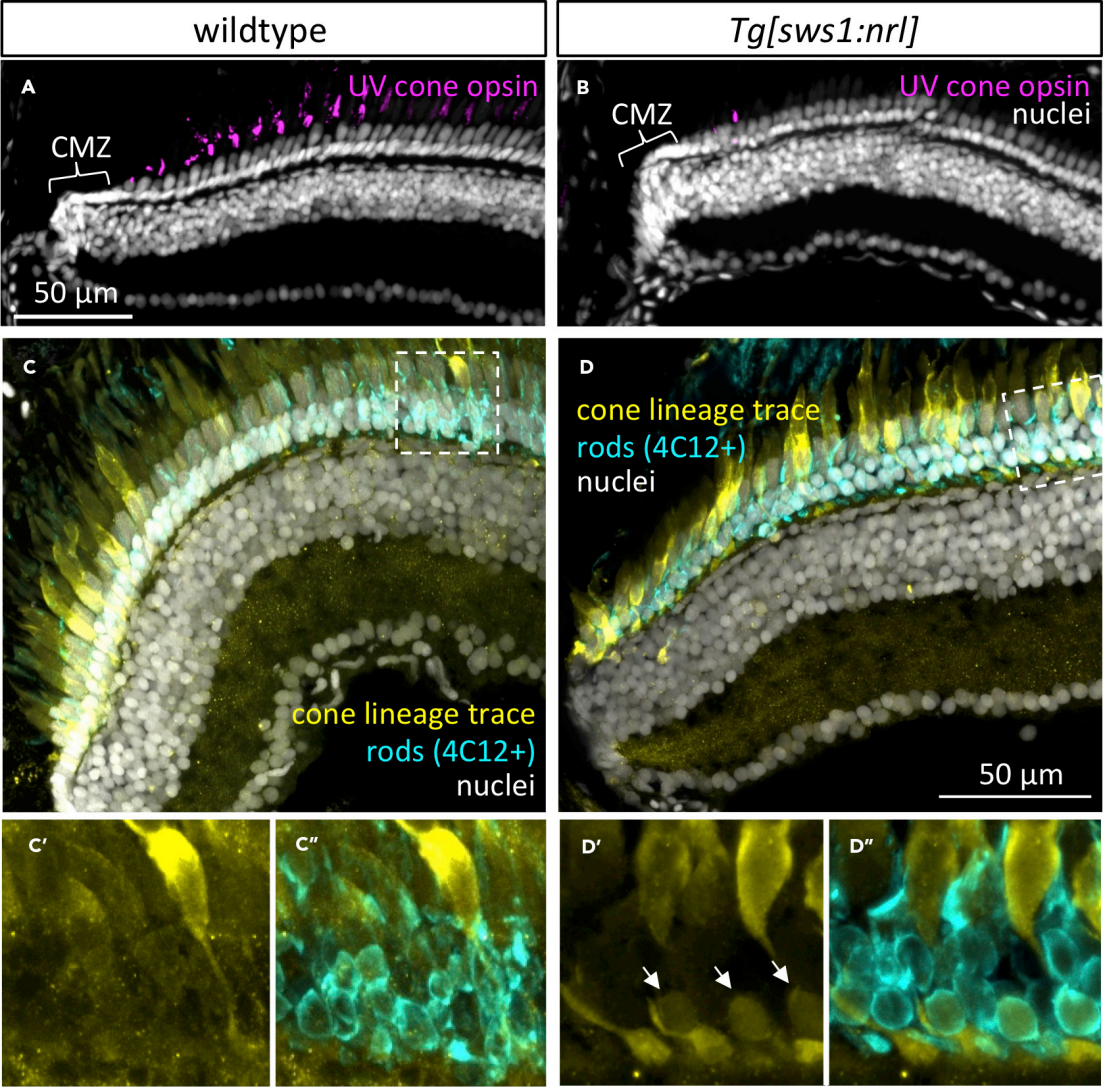
The Cone Cell Lineage Does Not Appreciably Contribute to Rod Production in Zebrafish but Inducing Ectopic Nrl Shows It has This Capacity (A) Adult zebrafish retina grows from proliferating cells in the ciliary marginal zone (CMZ), generating all cell types including regularly spaced UV cones. (B) Following ectopic expression of Nrl in differentiating UV cones, adult retina is mostly devoid of UV cones, except sparse newly born UV-opsin-positive cells near the CMZ. (A) and (B) are anti-UV opsin immunohistochemistry (magenta) with nuclear counterstain. (C) The adult zebrafish cone cell lineage gives rise to all cone cell types, and few other cell types are appreciably generated from that lineage, as detected by Cre-lox lineage tracing via a cone-transducin-α (*gnat2*) Cre driver line. No history of *gnat2* expression (yellow) is detectable in rod cells (4C12+), despite the lineage trace reporter being abundant in all other photoreceptors (cones). (C’) and (C″) are alternate views of dashed box outlined in (C). See also associated Figure S4. (D) The fate of UV-opsin-positive cells that are absent from mature retina in (B) includes their transmutation into rods. Note a subset of rod cells (arrows, identified as 4C12+ and with nuclei in the basal-most layer of the ONL) shows a history of *gnat2* expression (yellow), indicating they were generated from the cone lineage.

In adult transgenic zebrafish expressing zebrafish *nrl* in UV cones, UV cones were only detected near the ciliary marginal zone (CMZ) (a region of retinal growth that continues to produce new retina into adulthood Figures 3A and 3B). This could be due to the death of the UV cones shortly after they developed or due to their conversion to a rod cell fate accompanied by the cessation of UV opsin production. To investigate this, we produced genetically encoded Cre-recombinase lineage tracing tools to assess cone cell fates.

To follow the fate of UV cones that ectopically express nrl into adulthood, we used a paradigm of genetically encoded lineage tracing wherein all cells emanating from the cone lineage will permanently express fluorescent reporters regardless of their subsequent cell fate (Figures S4A and S4C). We drove transgenic expression of Cre recombinase under control of regulatory sequences for *gnat2* (cone transducin α) to induce Cre expression in all subtypes of developing cones (Kennedy et al., 2007; Suzuki et al., 2013). We bred this transgenic (Tg) line to two lox-mediated reporter lines, Zebrabow Pan et al. (2013) (Figure S4A) and ubi:Switch (Mosimann et al., 2011; Solek et al., 2017) (Figure S4C). In this cone lineage tracing line, zebrafish possessing wild-type *nrl* had no rods that were observed to originate from the cone cell lineage (i.e. none expressed *gnat2*:Cre-mediated fluorescent reporter; 8/8 fish with Ubi:Switch, 1/2 fish with Zebrabow, Figures 3C and S4E).

The fate of disappearing UV cones (the UV cones expressing ectopic Nrl in our Tg fish) was examined by incorporating these same cone lineage tracing reporter constructs into *Tg[sws1:nrl]* fish. Contrasting the results from wild-type zebrafish (Figure 3C), cone lineage tracing demonstrated that *Tg[sws1:nrl]* fish possessed rods expressing the cone lineage reporter (8/8 fish with Zebrabow) (Figures 3D and S4F, arrowheads). The characterization of cells from the cone lineage as being rods, in *Tg[sws1:nrl]* fish, was based on both the basal location of their nuclei within the outer nuclear layer (ONL) and their immuno-colocalization with the rod marker 4C12 (Figure 3D).

In 1 of 2 lineage trace zebrafish with wild-type *nrl*, using the Zebrabow paradigm, we noted a total of 27 lineage-trace-reporter-positive rods, in 5 clumps, in the oldest parts of the retina near the optic nerve head (Figure S4E); in the same fish, there was also expression of lineage reporter in other cell layers, including in some bipolar cells and ganglion cells. Considering the rarity of these rod cells and their presence in only a single fish (and only from one of two reporter lines), we suggest they are attributable to stochastic Cre-like DNA recombination events or spurious expression of the transgenic construct. The robust and abundant expression of cone lineage tracing reporter in rods only occurred in animals that also expressed Nrl in UV cones, suggesting that expression of Nrl in zebrafish UV cones is sufficient to reprogram them to a rod phenotype. These conclusions were supported in larval fish (6/6 retinas examined; Figure S4D) using the Zebrabow reporter line. This confirms that rod development in zebrafish proceeds in a straightforward manner that does not incorporate the cone lineage, unlike mice that use a circuitous route and produce many rod cells via the cone progenitor lineage (Kim et al., 2016).

### Nrl Is Dispensable for the Specification of Rods in Adult Zebrafish

**Figure 4:**
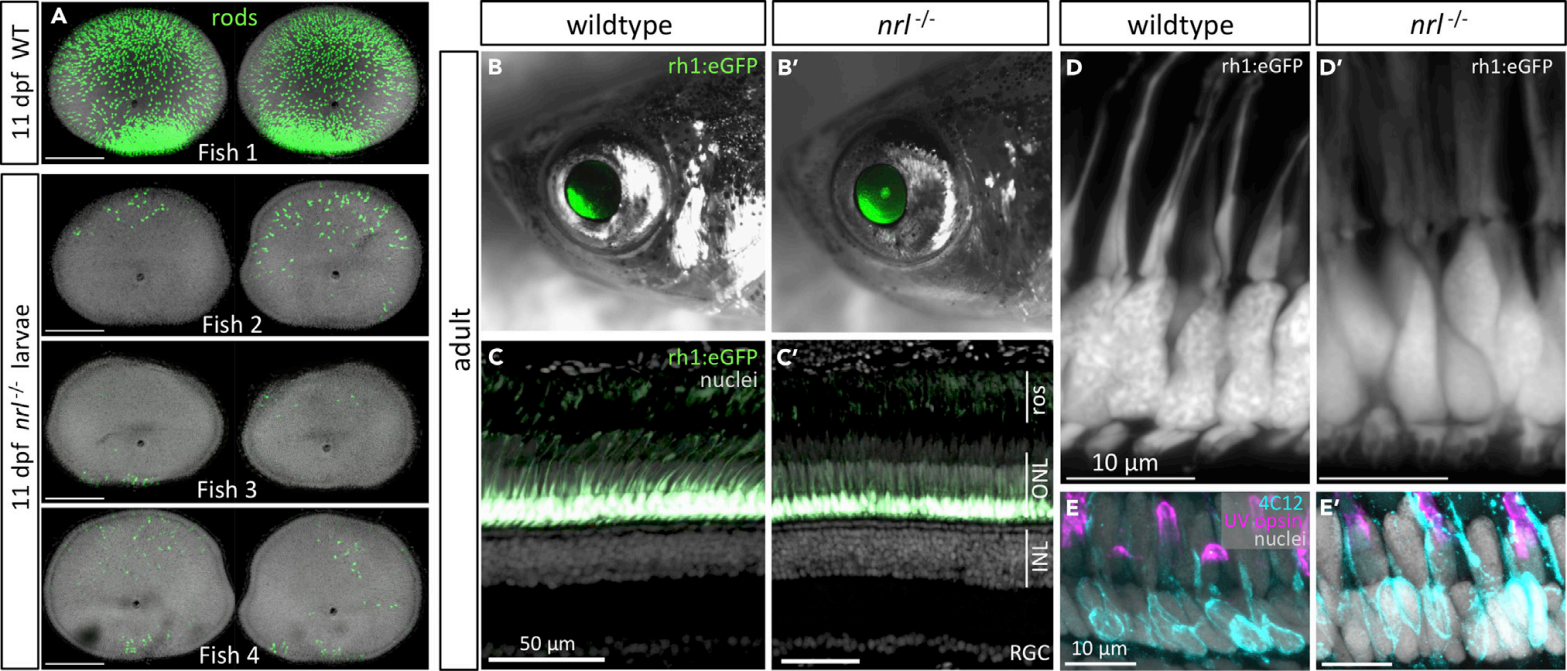
Nrl is Dispensable for Rod Specification in Adult Zebrafish (A) Monitoring for appearance of GFP-positive rods during ontogeny of *nrl*^−/−^ larvae directed our attention to 11 days postfertilization (dpf), where rods are sporadically detectable, but varied between individuals and between eyes of the same individual. Wild-type (WT) eyes at top with abundant rods provided for context. (B–D) Adult *nrl*^−/−^ zebrafish possess a large abundance of rods indistinguishable from WT, such that GFP-positive rods are obvious in intact animals (B) or retinal cryosections (C). Adult *nrl*^−/−^ retina showed normal distribution of rod outer segments (ros) apical of rod cell bodies in the outer nuclear layer (ONL, also in [D]). Inner nuclear layers (INLs) and retinal ganglion cell layers (RGCs) are overtly normal (quantified in Figures S5G and S5H). (E) Immunolabelling with rod-specific 4C12 and anti-UV-opsin confirms presence of rods and normal UV cones, respectively, in adult *nrl*^−/−^ retina.

To assess the impact of *nrl* loss on adult photoreceptors, we examined the photoreceptor composition of adult *nrl*^*−/−*^ mutant fish. Surprisingly, zebrafish adults with homozygous *nrl* mutation produce abundant rod photoreceptors (Figures 4B and 4C).

To affirm that the presence of rods in adult *nrl*^*−/−*^ fish represented a difference based on ontogenetic stage (rather than a stochastic difference between individuals) and to characterize when rods first appear in the *nrl*^*−/−*^ retina, we sought to assess individuals through their development. Breeding *Tg[rh1:eGFP]* into the *nrl*^*−/−*^ background allowed us to monitor for the appearance of GFP-positive rod cells in the eye of developing larvae and directed us to focus our characterization on 11 dpf. The benchmark comparator is wild-type larvae at 11 dpf, where retinas consistently had a large abundance of rods (thousands of rod cells per eye), including a concentration of rods in the ventral region and a large density of rods throughout all other retina regions (e.g. fish 1 at the top of Figure 4A). Retinas from *nrl*^*−/−*^ larvae at 11 dpf possessed a scattering of rods (~10–100 rod cells per retina, Figure 4A), contrasting younger *nrl*^*−/−*^ larvae where rods were never observed. Notably, the abundance and location of the rods varied considerably between individuals and between the two eyes within individuals (three representative examples displayed in Figure 4A, and such variation was apparent following examination of dozens of larvae). The stochastic nature of rod distribution in larval *nrl*^*−/−*^ fish is no longer apparent in adults, where the large abundance of rods is not different from wild-type animals (Figure 4C and quantified in Figures S5G and S5H). We estimate that rods can be produced using a mechanism independent of *nrl* beginning late in the ontogeny of larval zebrafish, stochastically appearing at about 11 dpf.

**Figure 5:**
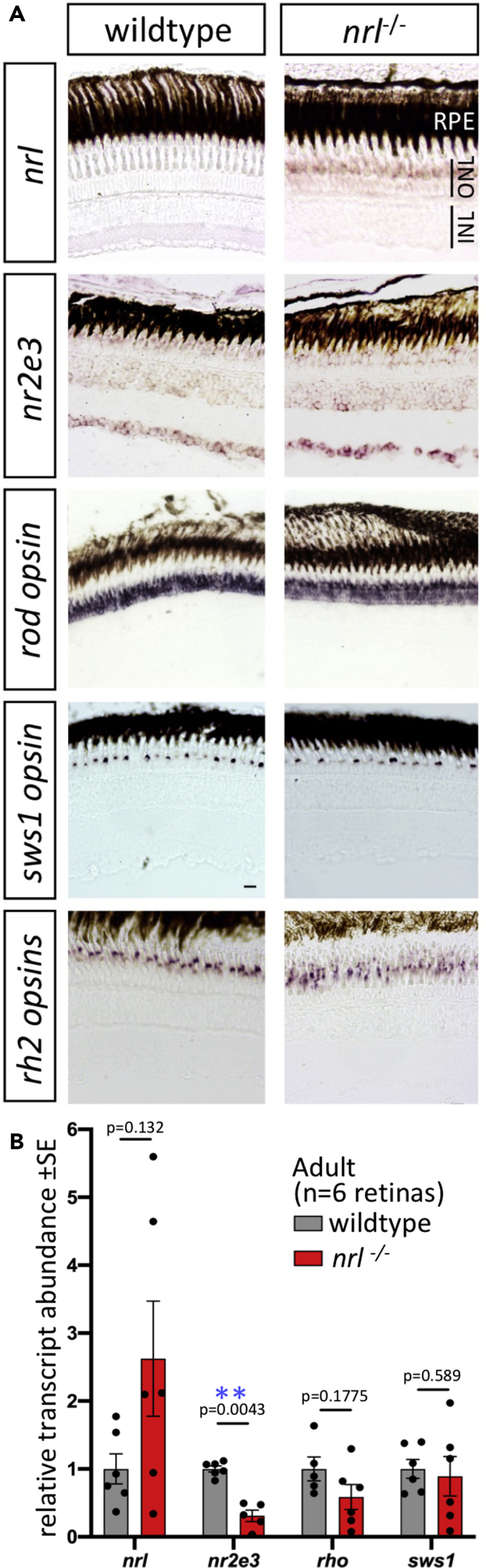
Adult Retina of Zebrafish *nrl*^−/−^ Mutants Show Changes in Abundance of Nrl Target Gene *nr2e3* and in *nrl* Itself (A) Gene expression determined by *in situ* hybridization on cryosections of adult zebrafish retina. Abundance of *nrl* transcript is higher in *nrl*^−/−^ frameshift mutant retina (confirmed in panel B), suggesting an auto-regulatory negative feedback loop controlling it own abundance. Assuming that *nrl* transcript location is unaltered in *nrl*^−/−^ mutants (despite aforementioned changes in its abundance), these data suggest expression of *nrl* is highly enriched in the outer nuclear layer, consistent with the site of phenotypes when Nrl protein is disrupted. Alterations to the abundance of *nr2e3*, a downstream target of Nrl, are equivocal when measured by *in situ* hybridization. The levels and distributions of transcripts encoding rod (*rh1*) and cone (*sws1* and *rh2*) opsins were not detectably different in *nrl*^−/−^ mutant retina compared to wild type. Scale bars represent 10 μm. (B) Transcript abundance in adult neural retina determined by RT-qPCR confirms an increase in *nrl* abundance in zebrafish bearing a frameshift null allele in *nrl*. A downstream transcriptional target of Nrl, *nr2e3*, was 70% reduced in abundance in *nrl*^−/−^ mutant retina compared to wild type (p < 0.01; n = 5–6 individuals per genotype). Opsin abundances in adult retinas were not markedly different between genotypes.

**Figure 6:**
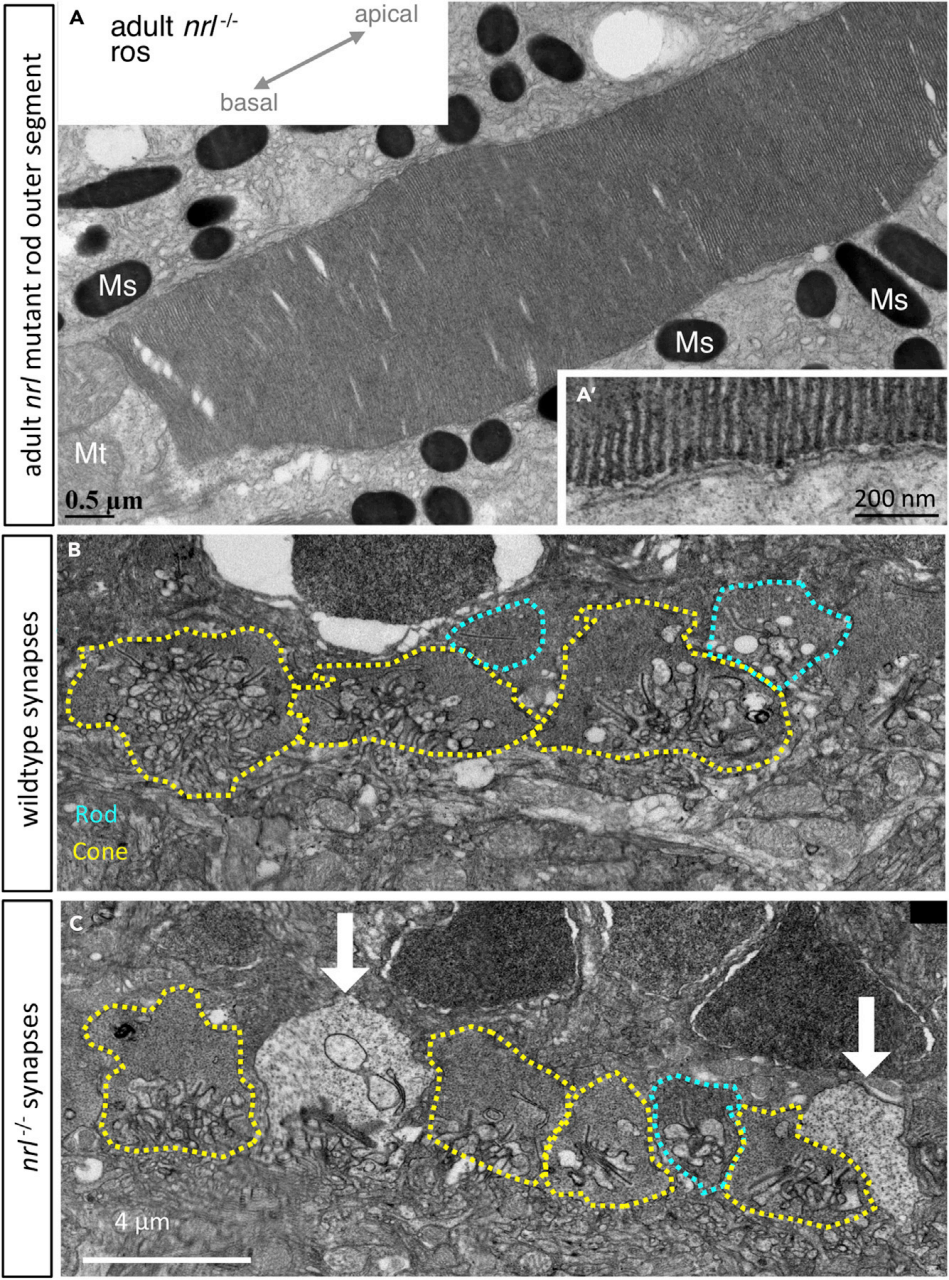
Rod Outer Segments of *nrl*^−/−^ Adult Zebrafish Appear Normal (A) Rods demonstrate the expected hairpin end of floating disks within the outer segment that are non-contiguous with the outer cell membrane (A′), diagnostic of rod cell identity (B) In wild-type adult zebrafish, photoreceptor synaptic terminals include rod spherules (teal dotted line) that are morphologically distinguishable from cone pedicles (yellow). (C) In *nrl*^−/−^ adult retina, the cone pedicles appear normal, and rod spherules appear normal but are very sparse; instead, electron-lucent terminals (white arrows) uniquely appear and may represent an Nrl-dependent defect in rod synapse maintenance. Various characters of photoreceptor terminals are quantified in Figure S6. Mt, mitochondria; Ms, melanosomes.

In adult retinas, all the rod photoreceptors markers that we tested each affirmed the presence of normal rods in *nrl*^*−/−*^ null zebrafish. Rod opsin transcript (*rh1*) is appropriately localized and abundant in adult *nrl*^*−/−*^ retina as determined by *in situ* hybridization on cryosections and by reverse transcriptase quantitative PCR (RT-qPCR) (Figure 5). Rods in adult *nrl*^*−/−*^ retina were also immunopositive for antibody 4C12 (Figure 4E’) and robustly express the rod-specific transgene *Tg[rh1:eGFP]* (Figures 4B and 4C). The morphology of the *nrl*^*−/−*^ rod cell bodies is normal at the level of confocal microscopy (Figure 4D), and the rod outer segment is normal at the level of ultrastructure (Figure 6A), showing freely floating disks indistinguishable from wild type.

An important aspect of concluding that Nrl is dispensable for rods in adult zebrafish is assessing whether any Nrl function is retained in adult *nrl*^*−/−*^ fish. A downstream effector of Nrl, *nr2e3*, was found to be ~70% less abundant in adult *nrl*^*−/−*^ retina (p = 0.0043; Figure 5B), consistent with Nrl immunoreactivity being abrogated in immunoblots from adult mutant retina (Figures 1G and S1D), and supporting that this is a null allele. Characterizing *nrl* transcripts in *nrl*^*−/−*^ retina further supported a potent disruption, insomuch that *nrl* transcript abundance was increased ~2.5 fold in adult *nrl*^*−/−*^ retina (not significant; Figure 5B); we infer that *nrl* may negatively regulate its own abundance (either directly or indirectly), and the absence of functional Nrl protein removes this negative autoregulation. The increased abundance of *nrl* transcript was also apparent via *in situ* hybridization on retina sections and offers good support that *nrl* expression is highly enriched in the outer nuclear layer (Figure 5A) and exactly consistent with the cellular location of the *nrl*^*−/−*^ phenotype (ONL containing the photoreceptor nuclei). In wild-type retinas, *nrl* transcript was not detected by *in situ* hybridization (Figure 5A). This appears to be an issue of sensitivity of our method, as *nrl* transcript is present in wild-type zebrafish rods (Nelson et al., 2008; Sun et al., 2018). The mutations in *nrl* also cause the abrogation of rod cells in larval fish and this exactly matches phenotypes predicted from *Nrl* knockout mice. Overall, disruption of downstream targets and loss of Nrl immunoreactivity in adult retina strongly match the prediction that our engineered frameshift mutation leads to a *nrl*^*−/−*^ null allele. Therefore, zebrafish are able to produce rod photoreceptor cells in their adult stages via a pathway independent of *nrl*.

### Lack of Obvious Phenotypes in Cones or Other Retinal Cells of Adult nrl^−/−^ Zebrafish

In light of the cone photoreceptor phenotypes in *Nrl* knockout mice, including an increase in sws1 cones (“S-cones”) (Mears et al., 2001) that we had also observed in larval *nrl*^*−/−*^ zebrafish (Figure 1), we characterized cone photoreceptors in adult *nrl*^*−/−*^ zebrafish. The highly organized cone mosaic in adult zebrafish retina, wherein cone spectral subtypes are positioned into repeating rows with high precision and fidelity (Allison et al., 2010; Engstom, 1960; Raymond et al., 1993, 1996; Vihtelic et al., 1999), ensures that any notable disruption to cone photoreceptors is obvious. No abnormal cone phenotypes were apparent in adult *nrl*^*−/−*^ retina. In particular, an overtly normal cone mosaic was apparent in adult *nrl*^*−/−*^ retina based on position of the nuclei (Figures S5A–S5F), with zpr1+ double cones flanking a single cone nucleus to form repeating pentameres of cone nuclei across the retina (Figures S5A and B). Lws cones and sws2 cones (red- and blue-sensitive cones) were spaced evenly across the adult *nrl*^*−/−*^ retina in their expected patterns, as detected by anti-lws-opsin and *Tg[sws2:mCherry]*, respectively (Figures S5C–S5F). Green-sensitive cones were distributed evenly across the adult *nrl*^*−/−*^ retina as determined by *in situ* detection of *rh2* opsin (Figure 5A). Immunolabelling and *in situ* detection of *sws1* opsin demonstrates adult *nrl*^*−/−*^ retinas possess a normal spacing of UV cones (Figures 4E and 5A, respectively). Tracing the cone lineage in adult *nrl*^*−/−*^ retina suggested a normal generation of cone subtypes (e.g. with no rod cells from the cone lineage) with the expected diversity of elaborated cone morphologies (Figure S5I).

Other retinal phenotypes associated with *Nrl* knockout mice, including rosettes or other defects in retinal lamination (Daniele et al., 2005; Samardzija et al., 2014), were not observed in retinas of *nrl*^*−/−*^ zebrafish (Figure 4C). Regardless, we explored for more subtle disruptions by quantifying cell abundances in radial retinal sections. In neither central retina nor peripheral retina is any difference in cell abundance detectable including for rods, cones, retinal ganglion cells, horizontal cells, or other inner nuclear layer cells (n = 6 fish per genotype; Figures S5G and S5H).

### Requirements for Nrl in Maintaining the Adult Zebrafish Rod Synapse

We noted that ultrastructural aspects of photoreceptor synaptic terminals were abnormal in adult *nrl*^−/−^ zebrafish. Cone pedicles were recognizable in wild-type and *nrl*^−/−^ retina due to their larger size, large abundance of synaptic ribbons, and somewhat electron-lucent appearance relative to wild-type rod spherules (Figure 6B). Ribbon length, vesicle density, and pedicle area in cones showed no dramatic differences between genotypes (Figure S6). However, normal rod spherules were rarely observable in adult *nrl*^−/−^ retina (Figures 6C and S6B), although when present they appeared normal in the aforementioned characters (Figures S6C–S6E). Unique to adult *nrl*^−/−^ retina, we noted a population of photoreceptor terminals that were broadly cone-like but extremely electron lucent such that we designated them as “white synapses” (Figure 6C, found abundantly in 4/5 *nrl*^−/−^ fish examined). Considering the discrepant large abundance of rod cells *vs.* the paucity of normal rod spherules in adult *nrl*^−/−^ retina, we infer these white synapses likely belong to *nrl*^−/−^ rod cells. Indeed confocal characterization of GFP-filled rod spherules (e.g. visible in bottom of Figure 4D) showed that the synaptic invagination was larger (perhaps more “cone like”) in *nrl*^−/−^ rod spherules compared to sibling rod spherules (Figure S6F). Compared to wild-type rod spherules, *nrl*^−/−^ white synapses had ultrastructure that was somewhat more cone like (larger size, longer ribbons, quantified in Figures S6C–S6E).

We found that the cone nuclei of wild-type and *nrl*^*−/−*^ adults were similar in chromatin texture and electrolucency (Figures S7C and S7D). However, we found that mutant rod nuclei had a mottled, though frequently still electron-dense appearance (Figures S7A and S7B). In the mouse, wild-type rods have a characteristic electron-dense arrangement of heterochromatin in the centers of their nuclei (Daniele et al., 2005; Corbo et al., 2010), whereas cone nuclei have a mixed arrangement of heterochomatin and euchromatin, leading to a mottled appearance. These two nuclear phenotypes are conserved in the zebrafish (Tarboush et al., 2012). Thus, *nrl*^*−/−*^ zebrafish rods may be thought of as exhibiting a cone-like chromatin appearance.

In sum, rod photoreceptors in adult *nrl*^−/−^ zebrafish are inferred to have electron-lucent and somewhat cone-like synaptic terminals, perhaps suggesting they have defects in synapse maintenance or differentiation. Rod photoreceptors in adult *nrl*^−/−^ zebrafish also have somewhat cone-like nuclear condensation at the ultrastructural level. However, rod photoreceptors in adult *nrl*^−/−^ zebrafish are specified as rod cells such that the diagnostic features of rod outer segments (rod opsin expression and disk ultrastructure) appear normal.

## Discussion

Our data describe a conserved role for Nrl early in zebrafish ontogeny that recapitulates its well-researched master regulatory role in mice, with Nrl being both necessary and sufficient for rod photoreceptor cell development. However, our data surprise by revealing that Nrl is dispensable for rod cell specification in adult zebrafish.

The rods present in adult *nrl* mutants have gene expression and ultrastructural differences compared to normal rods, including cone-like nuclei and synapses, indicating that *nrl* is involved in, but not solely responsible for, rod development in adult zebrafish. We also evaluated the capacity for zebrafish *nrl* to override the cone specification program in maturing UV cones and found that the erstwhile UV cones eventually adopted an unambiguous rod-like phenotype, indicating a conserved capacity for zebrafish *nrl* to induce rod characters. Indeed, this capacity to induce rod characters was indistinguishable in transgenic fish expressing mouse or zebrafish homologs of *Nrl*.

Appreciating the mechanisms whereby the transcription factor NRL specifies rod *vs.* cone photoreceptor cells is foundational knowledge to at least five disparate fields of evolutionary and biomedical research. First, this work will be of interest to theorists seeking to understand the mechanisms of how novel cell types arise over evolutionary time. Proposals for these mechanisms have benefited from a focus on photoreceptors, which have rapidly diversified early in vertebrate evolution, and the role of Nrl in mouse photoreceptor specification has been an instructive case study in cell type evolution (Arendt et al., 2016). The existence of Nrl-independent phenotypes in zebrafish rods may indicate an additional cell type diversification among rods which can be further mined for insight. Second, mutations in the *NRL* gene (along with its downstream effector *NR2E3*) are causal of blinding disorders that remain unchecked (Bessant et al., 1999; Nishiguchi et al., 2004). Third, *Nrl*^*−/−*^ knockout mice have broadly imposed themselves upon animal modeling of ophthalmology, often being used as a (relatively artificial) proxy for the cone-rich macular region of human retina. Fourth, gene therapy strategies that disrupt *NRL* show substantial promise in mouse models as a cure for retinitis pigmentosa and other rod degenerative disease – a mechanism to save degenerating rods by converting them into a cone-like state (Yu et al., 2017). Finally, stem cell therapies to repair vision loss have demonstrated great potential but must now overcome the hurdle of regenerating cones (rather than rods) to repair daytime and high-acuity vision, and *NRL* is at the heart of the gene network driving this cell fate switch. Appreciating NRL function beyond nocturnal mice begins to fill a substantial and influential knowledge gap: It is surprising that the function of Nrl had remained untested in zebrafish, despite zebrafish emerging as the premier genetic model of vertebrate photoreceptor development and regeneration.

Moreover, we and collaborators recently proposed that evolution in Nrl's function and/or regulation are prime candidates for a proximate mechanism in the evolutionary success of early mammals as they adapted to survive the nocturnal bottleneck (Kim et al., 2016). Comparative lineage tracing between mice and zebrafish demonstrated that a majority of rod cells arise from an unexpected source in mice – the cone progenitors – whereas cone lineages only gave rise to cones in zebrafish. The lineage tracing results in mice were bolstered by analyses of transcripts and protein in developing rods that revealed vestiges of sws1 cones (Kim et al., 2016). That taxonomic comparison suggested a developmental innovation had contributed to evolving the rod-rich retina of mammals, and we proposed changes at the Nrl locus of early mammals as a potential driver of this adaptation. The proposal aligns well with previous data showing ectopic expression of Nrl in mouse retinal progenitors as being sufficient to drive the rod cell fate (Oh et al., 2007).

### Speculative Relevance of nrl for Vertebrates Adapting to Dim Light Visual Tasks

Photoreceptor ratios and diversity have shifted greatly in some vertebrate lineages, reflecting the importance of updating visual capacity to match emerging ecological needs over evolutionary time (Lamb, 2013). This has resulted in species losing one or more of the ancestral photoreceptor types, interconverting photoreceptor types, and/or shifting rod:cone ratios to adjust to changing levels of illumination in new habitats.

The conversion of rods to cones or cones to rods was described by Walls, who termed it photoreceptor transmutation (Walls, 1942). His evidence for this phenomenon derived mostly from comparing species of geckos and snakes, although there is now evidence that this has occurred in numerous lineages. The Tokay gecko is nocturnal but has no rods and instead has adapted cones (Yokoyama and Blow, 2001) and cone-specific phototransduction machinery (Zhang et al., 2006) to replicate rod physiology. Garter snakes have lost green cones but converted rods to cone-like physiology to play a green cone-like role (Schott et al., 2016); in another snake, a rod which had previously gained a host of cone-like qualities appears to be underway toward evolving a rod-like physiology once more (Bhattacharyya et al., 2017). The deep-sea pearlside fish has both rods and cones, but all cones have been converted to a rod-like physiology, essentially producing an all-rod retina (de Busserolles et al., 2017). The tiger salamander has evolved a second type of rod, the so-called green rod, which expresses Sws2 opsin and is sensitive to blue light (Isayama et al., 2014; Mariani, 1986; Zhang and Wu, 2009), although these salamanders retain a dedicated Sws2-expressing cone as well. Photoreceptor transmutations appear to be a viable solution to increasing or shifting photoreceptor diversity as needed over evolutionary time. We and colleagues recently demonstrated evidence that a cone-to-rod transmutation may have occurred in ancient mammals and proposed that ancient Nrl may have been competent to recruit cones to the rod phenotype (Kim et al., 2016), which the present study supports.

Recent work on Atlantic cod provides additional evidence that nrl-independent rod specification may occur in nature (Valen et al., 2016). Valen and colleagues characterized the ontogeny of photoreceptors in the cod, which lives for 30 days as a pre-metamorphic cone-only larvae and develops its first rods during metamorphosis between 30 and 53 days posthatching. Valen and colleagues performed comparative RNAseq experiments from various time points, and nrl transcripts are not abundant beyond the first days of life in these fish. Later, nrl transcript abundance actually decreases at the time when rod cells are most rapidly generated (37 days posthatching). Nrl and the other Maf family transcription factors have been implicated in lens development (Reza and Yasuda, 2004; Coolen et al., 2005), and our mutants display lens development defects (Figure S2). It is possible that, in the Atlantic cod, nrl has played a role in lens or other development only and that cod have lost larval nrl-mediated rod production. This suggests that at least in fish, the strict requirement for nrl in specifying rod photoreceptors relaxes during ontogeny.

### Speculations on How Adult *nrl*^*−/−*^ Zebrafish Produce Rods

An outstanding question remains: How are rods produced in the adult nrl mutants? Speculations include partial redundancy with similar genes or the occurrence of transcriptional adaptation that has recently accounted for surprising results following engineering of frameshift mutations. Our data, including an exploration of Maf genes in the *nrl*^*−/−*^ mutants, do not support these speculations (though they also cannot rule them out). While no *nrl* paralogs have been detected in zebrafish, *nrl* belongs to the Maf family of transcription factors that are represented in the genome by two paralogs of each *maf*, *mafa*, and *mafb*. Moreover, *MafA* has been implicated in the development of rods in avian retinas where *nrl* has been apparently lost (Enright et al., 2015). Alternatively, mechanisms have been proposed which explain how decaying mutant mRNAs may stimulate expression of other genes with similar sequence to their own (Serobyan et al., 2020; El-Brolosy et al., 2019; Ma et al., 2019), termed “transcriptional adaptation" or "genetic compensation response” depending on the mechanism. The data in *nrl*^*−/−*^ mutants are not consistent with this mechanism, in that the *nrl* transcript abundance is not lower in the mutants (indeed it is perhaps increased, Figures 5B and S8). Moreover, the observation that phenotypes in *nrl*^*−/−*^ mutants (absence of rods) depend on ontogeny also requires a more challenging speculation that transcriptional adaptation mechanisms vary over ontogenetic stage.

We assessed these ideas by quantifying the relative abundance of all the zebrafish maf genes throughout development (assessing whole larvae, Figure S8), especially including the time period where *nrl*^*−/−*^ mutant rods were initially produced. We did not find changes in transcript abundance consistent with transcriptional adaptation. Only *mafb-a* and *mafb-b* had shifted transcript abundance in the mutants, with *mafb-b* strongly and significantly more abundant at 8 dpf in mutants, and both *mafb-b* and *mafb-a* slightly though significantly more abundant at 9 dpf in mutants. However, these transcripts were not more abundant at later points in development as would be required in transcriptional adaptation. Quantifying *mafbb* in adult neural retina confirmed it was not more abundant in *nrl*^*−/−*^ mutants (Figure S8). Future work might focus on more sensitive detection approaches, isolation of particular tissues or cells, or examining the chromatin state induced by the various mafs since this appears to shift to a more permissive state during genetic compensation response. Overall, these data speak against potential artifacts of genetic compensation in *nrl*^*−/−*^ mutants, leaving biological speculations that the gene regulatory network surrounding *Nrl* (e.g. *Crx* and *Otx* genes, etc.) could be the source of differences in zebrafish *vs.* mouse rod photoreceptor specification.

A difference in rod photoreceptors between mice and zebrafish is that zebrafish rods express two paralogs of the rod opsin gene *rh1* (Morrow et al., 2011; Sun et al., 2018), whereas mammals possess a single copy. Our analyses were designed to measure the expression of transcript *rh1-1* (*rho*), but we cannot rule out that *rh1-2* (*rhol*) transcripts might have also been detected by our qPCR and riboprobe reagents. Parallel to this, our riboprobes do not distinguish between the four paralogs of zebrafish *rh2* opsins (MWS or green-sensitive opsins, with gene names *opn1mw1* thru *opn1mw4*). This is also a matter of future interest, as paralogs *rh2-1* and *rh2-2* are expressed early in development, and onset of *rh2-3* and *rh2-4* expression occurs later (by 16 dpf) (Takechi and Kawamura, 2005; Mackin et al., 2019) at a time point somewhat coincident with the appearance of *nrl*^*−/−*^ rod photoreceptors.

Moreover, differences in the rod cell lineage between mice and fish also inspire speculations on how rods might be produced in *nrl*^*−/−*^ mutant zebrafish. While photoreceptor development is complete shortly after birth in mammals, rods are continually generated throughout the lifespan of fish (Allison et al., 2006a, 2006b; Fernald, 1990; Raymond and Rivlin, 1987; Hagedorn et al., 1998). Fish appear to have two major routes for rod production, according to their developmental stage. In early development, rods come from photoreceptor precursors. In adult fish, rods are continually generated throughout the retina as it expands, principally from an endogenous stem cell population that includes Müller glia (Bernardos et al., 2007; Lenkowski and Raymond, 2014). The Müller glia may typically produce only rods in healthy animals, but after retinal damage, they are capable of producing all forms of retinal cells, including cones (Hagerman et al., 2016; Fraser et al., 2013), and thus, differentiation frameworks must be in place to govern the production of specific cell types, including rods in a healthy animal. The fish ability to derive new photoreceptors from endogenous glia in adults is exciting and a topic of medical research, but it remains unclear whether the mechanisms that control photoreceptor differentiation in this pathway are the same as in development. The data presented herein that zebrafish rod specification requires *nrl* only in early ontogeny provide a striking parallel to the literature on Müller-derived rods that become prominent late in ontogeny. Further work is warranted to assess if cells derived from the endogenous stem cells of the Müller glia lineage can specify rods independent of Nrl.

### Lineage Tracing of Adult Rod Photoreceptors Using *gnat2*-Induced Reporter in the Absence of Ectopic *nrl* Expression

We previously demonstrated that larval zebrafish rods do not have a history of *sws1* expression (Kim et al., 2016), using a Gal4/UAS-derived technology. Zebrafish silence the UAS promoter as they age and over generations, precluding the use of that arrangement of genetically encoded lineage tracing constructs for this study. Here, we found that another cone gene, *gnat2*, also did not report expression in any rod in zebrafish larvae using a Cre/Lox lineage tracing system (Figure S4D), consistent with and extending the previous study concluding that larval zebrafish rods do not exhibit a history of expressing cone genes. While the *gnat2:cre* lineage tracer robustly reported rods in conjunction with ectopic *nrl* expression in UV cones (Figures 3 and S4), we noted 5 clusters of lineage-traced rods, along the length of a retinal section from CMZ to optic nerve head, in one animal without transgene-induced ectopic *nrl* expression (Figure S4E, arrowheads). In animals of both genotypes, we noted occasional labeling of other cell types (e.g., Figure S4F, a bipolar cell). We consider it likely that the 5 clusters of lineage traced rods represent 5 clonal populations of rods with one-time spurious expression of *gnat2:cre* during their development.

### Zebrafish *nrl*^*−/−*^ Rods and Cone-Rod Transmutations in Nature

Close examination by electron microscopy of the adult *nrl*^*−/−*^ rods suggested that the mutants do not make typical rod synapses; across two mutant animals, we found a single synapse that was clearly rod-like: electron dense relative to neighboring cone photoreceptors, a single synaptic ribbon that was longer than nearby cone ribbons, and placed either between cone synapses or positioned slightly scleral within the synaptic layer, consistent with previous zebrafish synapse characterization (Tarboush et al., 2012). The remaining synapses appeared to be either cone synapses, or “white” synapses (in 4/5 animals assessed). The “white” synapses were positioned like cones within the synaptic layer, interspersed among cone synapses. There were not enough of them to fully account for the lack of obvious rod synapses; if they belonged to the rods, then other synapses, perhaps more overtly cone-like synapses, likely did as well. This is reminiscent of the synapses of lamprey photoreceptors. At least one species of lamprey have cells with the physiological characteristics of rods: the ability to respond reliably to single photons of light, sluggish responses to stimulation relative to the more cone-like lamprey photoreceptors, and the ability to send their signals to the cone-like photoreceptors (Asteriti et al., 2015; Morshedian and Fain, 2015). However, lamprey rod-like cells also have cone-like characteristics; *Mordacia mordax*, a nocturnal lamprey with a single photoreceptor with rod-like physiology, has plasmalemma invaginations in the outer segment that mean it does not have rod-like free-floating membrane discs but instead has a cone-like morphology (Collin and Pottert, 2000; Collin and Trezise, 2004). *Petromyzon marinus* has rod-like synapses associated with the physiologically rod-like cell, but these can have up to 4 synaptic ribbons, and the ribbons do not appear to differ in length between photoreceptor types (Dickson and Graves, 1979). Outside lamprey, the teleost deep-sea pearlside *Maurolicus muelleri* has photoreceptors deemed “true” rods (rhodopsin expressing; minority) and rod-like cones (green opsin expressing; majority), and the synapses of the rod-like cones were smaller (rod like) but had multiple synaptic ribbons per terminus (cone like) (de Busserolles et al., 2017). It was proposed that the rod-like cones of *M. muelleri* are “transmuted” cones, in the sense of Walls (Walls, 1942).

The mottled chromatin texture of the *nrl*^*−/−*^ mutant rod (Figure S7) is consistent with the mottled chromatin of the cone-like photoreceptors produced in the *NRL*^−/−^ mouse (Daniele et al., 2005) and reminiscent of the texture of zebrafish cones (Figure S7, and (Tarboush et al., 2012)). The dense heterochromatin of wild-type rods is possibly a solution to improve photon transmission and thus increase sensitivity of rods in dim light conditions and is particularly consistent among nocturnal animals (Solovei et al., 2009). We note the similarity of the *nrl*^*−/−*^ zebrafish rod nuclei, which appear to be rods despite ultrastructural cone-like similarities, to the nuclei of the rod-like photoreceptors in *M. mordax* (see Figures 7A and 7B of Collin and Pottert, 2000 (Collin and Pottert, 2000)), and the nuclei of the rod-like photoreceptor of *P. marinus* are clearly mottled and nearly indistinguishable from the cone-like photoreceptor nuclei (see Figures 12 and 13 of Dickson and Graves, 1979). In the avian lineage, which unambiguously has rod-like photoreceptors but which has also lost NRL, electron microscopy of the common buzzard (*Buteo buteo*) suggested that all photoreceptors have mottled chromatin, although this was not explored in depth (El-Beltagy Ael, 2015). In the deep-sea pearlside *M. muelleri*, the nuclei of both the true rods and rod-like cones had obvious mottled chromatin (de Busserolles et al., 2017). It was not reported whether *M. muelleri nrl* was present in the transcriptome data. Furthermore, it is possible that examination of photoreceptor synapses and nuclei could be used to probe for cryptic or suspected transmutation events.

Thus, there are numerous examples of animals bearing rods with some cone-like aspects, which align with scenarios where an originally cone-like cell may have become rod like. This would imply that the *nrl*^*−/−*^ zebrafish rods use cone machinery or are derived incompletely from cells that started as cones. Future work might assess whether the *nrl*^*−/−*^ zebrafish rods deploy cone phototransduction machinery, as cone-rod transmutation events seem to leave various species using a mixture of cone and rod proteins for phototransduction. Our initial examination of this, with the gnat2:cre lineage tracing construct, did not label zebrafish *nrl*^*−/−*^ rods, suggesting they do not derive from post-mitotic cone-fated cells, and furthermore suggesting that these rods do not employ gnat2 (a cone phototransduction gene); however, many other cone phototransduction genes ought to be assessed to determine whether there is a blended phenotype between cones and rods.

In summary/conclusion, classic interpretation is that NRL is the absolute master regulator of the rod photoreceptor cell fate; we demonstrate here that this role is deeply conserved, and yet not completely conserved, in an intriguing manner outside of mammals. Despite the canonical requirement for NRL being apparent in larval zebrafish, adult zebrafish lacking Nrl were shown to specify and produce an abundance of rod photoreceptors. The *nrl*^*−/−*^ rods show only subtle ultrastructural and transcriptional differences from wild-type rods. The unexpected tolerance of adult zebrafish rods for the absence of nrl suggests that larval, not adult, zebrafish rods are best suited for biomedical modeling of mammalian rod dystrophies.

Across ontogeny, nrl expression is sufficient to induce a robust rod phenotype in developing cone photoreceptors, suggesting that cone photoreceptors remain competent to transmute to rods throughout the life of the animal. This has implications for the mechanisms enabling cone-to-rod transmutations found in various vertebrate lineages, including in early mammals.

Zebrafish retain ancestral vertebrate retina traits that have been lost in mammals, including the original complement of 4 cone subtypes and 1 rod. The unexpected difference between fish and mouse in nrl requirements reinforces the need to explore the developmental genetics of retinal cells across a range of vertebrates if we are to build a comprehensive understanding of retinal development and evolution.

### Limitations of the Study

Rods are specified and abundant in adult *nrl*^*−/−*^ zebrafish, though outstanding questions concern the extent to which these rods are functional. The abnormal rod spherules in these mutants suggest that physiological deficits at the first synapse are likely. Nrl in other species influences expression of various genes, and so deficits in phototransduction are also possible. This gap in our characterization limits the depths of our conclusions.

We speculated in the discussion above how it may be that rods can be produced in adult *nrl*^*−/−*^ zebrafish, including why our data do not suggest transcriptional adaptation as a likely mechanism. Despite this, without further investigation, we cannot formally dismiss those alternative explanations at this time. Similarly, several lines of evidence strongly support that the *nrl*^*−/−*^ mutant animals represent a null allele; however, the absence of detectable gene products (including Nrl protein) cannot formally rule out their existence; future studies might complement this work with *nrl*^*−/−*^ mutant fish that lack the entire gene.

### Resource Availability

#### Lead Contact

Further information and requests for resources and reagents should be directed to the corresponding author.

#### Materials Availability

All unique/stable reagents generated in this study are available to qualified researchers via contacting the Lead Contact.

#### Data and Code Availability

The published article includes all data sets generated or analyzed during this study.

## Methods

All methods can be found in the accompanying Transparent Methods supplemental file.

## Supplemental Information

Document S1. Transparent Methods, Figures S1–S8, and Table S1
