# Supplementary material for: Nrl Is Dispensable for Specification of Rod Photoreceptors in Adult Zebrafish Despite Its Deeply Conserved Requirement Earlier in Ontogeny
Source: iScience. 2020 Nov 15;23(12):101805. doi: 10.1016/j.isci.2020.101805 (PMC7702016; doi:10.1016/j.isci.2020.101805)
Supplement: Document S1. Transparent Methods, Figures S1–S8, and Table S1 [file mmc1.pdf]

iScience, Volume *23*

Supplemental Information

Nrl Is Dispensable for Specification of Rod Photoreceptors in Adult Zebrafish Despite Its Deeply Conserved Requirement Earlier in Ontogeny

A. Phillip Oel, Gavin J. Neil, Emily M. Dong, Spencer D. Balay, Keon Collett, and W. Ted Allison

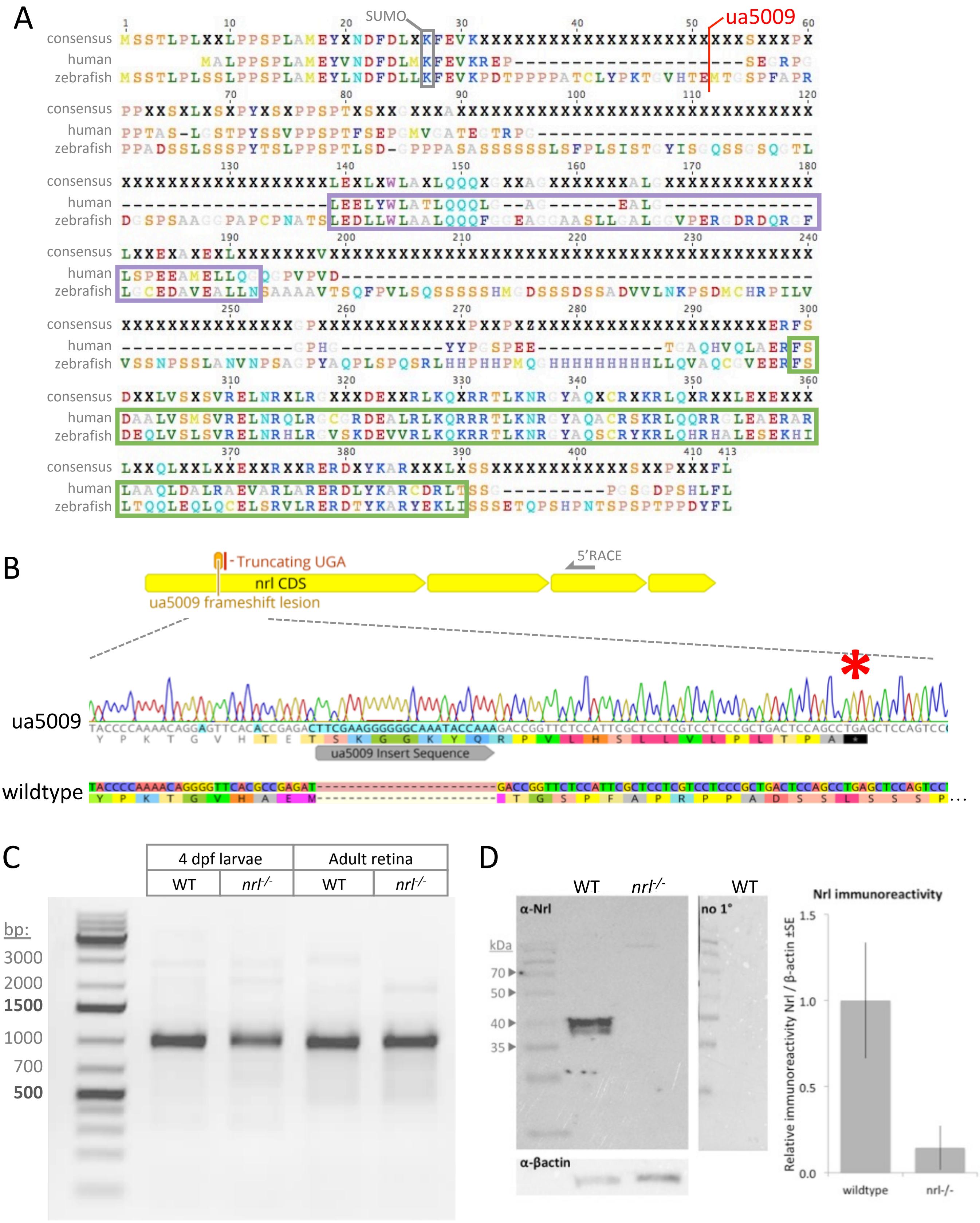

Figure S1. **Most aspects of zebrafish Nrl protein are conserved with mouse… (legend on next page)**

Figure S1. Most aspects of zebrafish Nrl protein are conserved with mouse and human Nrl, and the key protein domains are predicted to be absent in *nrl*$^{-/-}$ frameshift mutants. Relates to Figure 1 and provides expanded views of data therein. **A.** Alignment to human Nrl reveals that the zebrafish Nrl homolog has insertions that make it relatively longer, but all key protein domains are recognizable. Maf N-terminal region (Maf_N) and basic leucine zipper DNA-binding (bZip) Domains are outlined and colour-coded purple and green, respectively as per Figure 1B. Both these domains are lost in *nrl*$^{-/-}$ allele ua5009 where an insertion (see panel B) creates a frameshift and therefore abrogates normal translation of Nrl after residue 51. An experimentally-validated SUMOylation site at residue K20 of human or mouse Nrl is perfectly conserved in zebrafish (residue K27 and surrounding residues in zebrafish Nrl), and likely accounts for the doublet band of Nrl appearing on immunoblots of wildtype retina (panel D). **B.** CRISPR/Cas9 engineered *nrl* allele ua5009 is a frameshift (23 basepair insertion) near the beginning of the first coding exon of the *nrl* gene. The frameshift leads to a termination codon (*) shortly after the insertion. This truncation is predicted to eliminate the recognizable protein domains of Nrl (schematized in Fig. 1B and S1A) and is predicted to be a null allele. **C.** Characterizing the *nrl* transcript in *nrl*$^{-/-}$ mutants shows no evidence of abnormal splicing, arguing against any cryptic exons being incorporated, and thus does not support any confounds to the prediction of a null allele. All *nrl* transcripts are amplified, regardless of their 5' content, using 5'RACE (Random Amplification of 5' cDNA Ends) with a primer positioned in the third coding exon (schematized in top right of panel A). Transcripts from wildtype (WT) and *nrl*$^{-/-}$ mutant tissues showed no evidence of disparities between genotypes, in either larvae or adult retina. The identity (mutant vs. wildtype) of the transcripts was confirmed by sequencing. This is an expanded view of data in Fig. 1H. **D.** Nrl protein is lost in adult *nrl*$^{-/-}$ mutant retina. Blots are an expanded view of data in Fig. 1G, and additionally demonstrate lack of signal development when primary antibody is excluded (right side). Histogram displays quantification of Nrl immunoreactivity relative to β-actin for n=3 individual fish of each genotype. Doublet band of immunoreactivity is reminiscent of blots of mammalian Nrl where mutagenesis has demonstrated this represents a post-translational SUMOylation; the SUMOylation site is perfectly conserved in zebrafish Nrl (Fig. S1A).

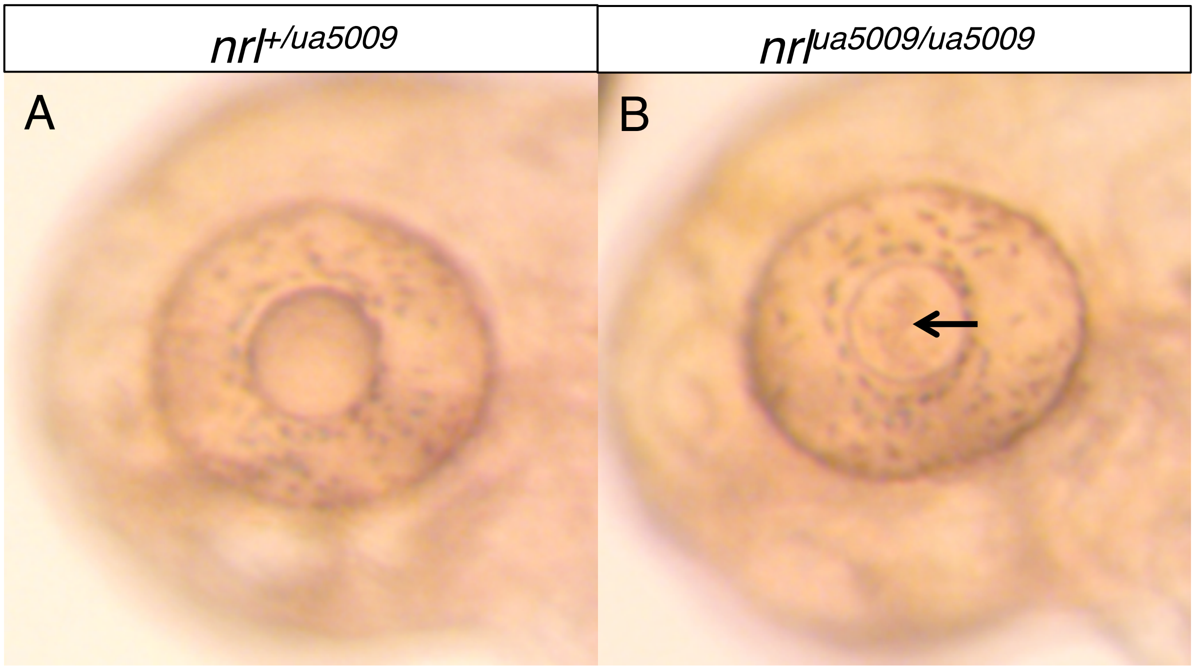

Figure S2. Proper zebrafish lens development requires *nrl*. Relates to Figure 1. A small inclusion is visible in the lens of $nrl^{-/-}$ larvae, and it remains detectable in adults (Fig. 4B').

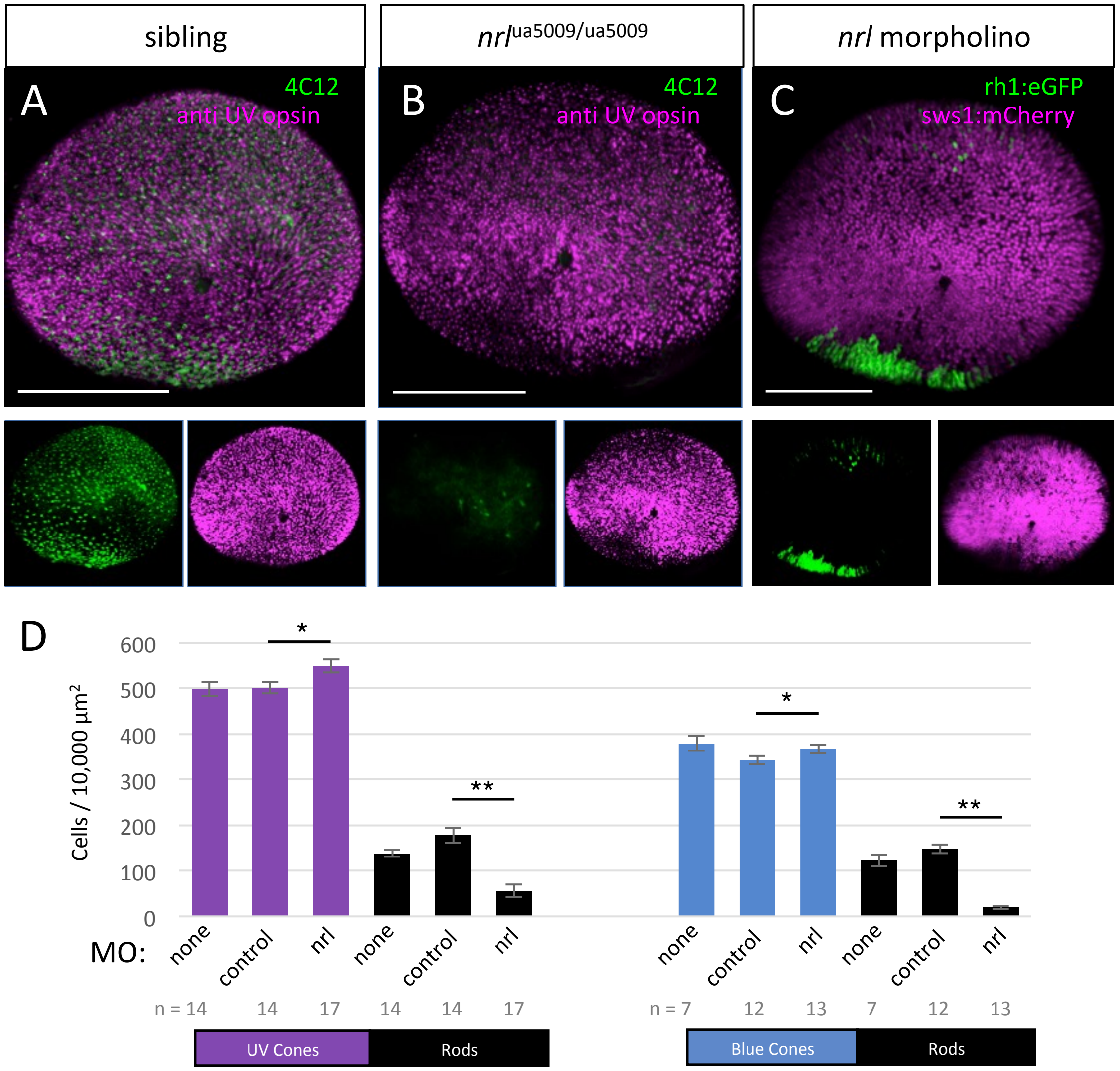

Figure S3. Morpholino knockdown of *nrl* phenocopies the *nrl* mutation, revealing drastic reduction of rod cells and increase in UV cones relative to wildtype. Relates to Figures 1. Wholemount retinas from 4dpf zebrafish, *en face* view (similar to Fig. 1C) with individual channels displayed below the merged channels. (A,B) green is 4C12 rod immunolabeling, while magenta is 10C9.1 UV cone immunolabeling; in panel C, rods express GFP from *Tg[rh1:GFP]* and UV cones express nfsb-mCherry (pseudocoloured magenta). **C.** 10ng of splice-blocking morpholino, targeting the first exon-intron boundary of *nrl* transcript, was injected into *nrl*$^{+/+}$ zebrafish and sharply reduced the abundance of rod photoreceptors in the whole retina. **D. Rod and UV cone cell abundances change following *nrl* knockdown by morpholino**. Splice-blocking morpholino against *nrl*, or an equivalent amount of standard control morpholino, was injected into wildtype *nrl*$^{+/+}$ zebrafish with the indicated fluorescent markers and rods and UV cones, or rods and Blue cones, were quantified within a 100 x 100 μm box positioned just dorsal to the optic nerve head. Rod abundance was substantially lower in nrl morphants relative to standard control-injected larvae, and a small (10%) but significant increase in UV cone abundance was observed. Morpholino injection decreased blue cone abundance, but less so with *nrl* morpholino compared to control morpholino. * is p<0.05, and ** is p<0.01 by Mann-Whitney U. n=number of individual larvae. Scale bars are 100 µm.

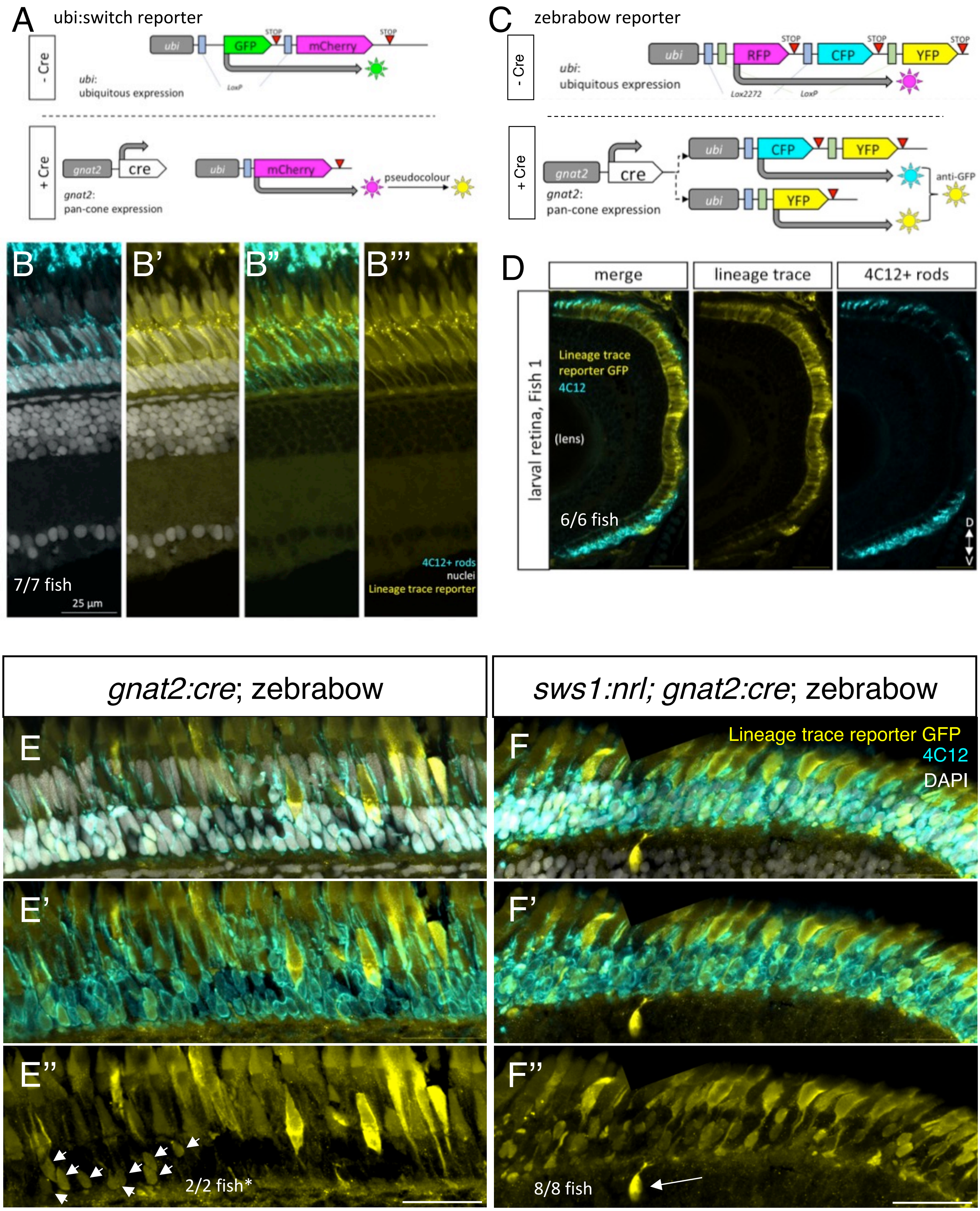

Figure S4. In wildtype zebrafish the cone photoreceptor lineage…(legend on next page)

Figure S4. In wildtype zebrafish the cone photoreceptor lineage does not give rise to rod cells, though ectopic expression of Nrl shows the cone lineage has this capacity if artificially induced. Relates to genetically encoded lineage tracing in Figure 3C & D. **A.** Schematic of lineage tracing elements in ubi:switch transgenic zebrafish. In the absence of Cre recombinase, all cells in these fish express GFP (top). Cells of the cone lineage were engineered to express Cre recombinase (bottom, driven by promoter from the *gnat2* gene encoding cone-transducin-α) and thus all cells from the cone lineage permanently edit the reporter DNA to express mCherry protein (pseudocoloured to yellow). In sum, all surviving cells of the cone lineage, and all their progeny, express mCherry (yellow) regardless of their subsequent cell fate. **B.** The cone lineage in adult zebrafish retina gives rise only to cone photoreceptors, and no lineage-tracer-positive cells were found to co-localize the rod cell marker 4C12 in 7/7 animals. **C.** Schematic of zebrabow lineage tracing reporter is conceptually equivalent to panel A and gives rise to fluorescent protein (again pseudocoloured yellow) in all cells derived from the cone lineage. Differences to the ubi:switch reporter are that in the absence of Cre all cells of the body express red fluorescent protein (RFP) and the presence of Cre permanently switches cells to express a mixture of cyan and green fluorescent protein (CFP & YFP) that are detected with anti-GFP immunohistochemistry and pseudocoloured yellow. All cells of the cone lineage and their progeny fluoresce with reporter colour (yellow) regardless of their subsequent cell fate. **D.** In larval zebrafish the cone lineage has high fidelity towards generating only cone photoreceptors, and no rod cells (detected by immunomarker 4C12) co-localize the zebrabow cone lineage tracer. Results consistent in 6/6 larvae at 5 dpf (days post-fertilization). **E.** Adult wildtype ($nrl^{+/+}$) fish bearing zebrabow cone lineage tracing broadly confirm results in panel B showing cone lineage labelling in 2/2 fish, *though in 1 of 2 examined retinas small clusters of lineage+ rods were observed; The largest such cluster (nine lineage reporter-positive rod cells, arrows) of this animal is shown, and 27 such cells were observed in five clusters, thus representing a tiny minority of rod cells and perhaps an artefact of stochastic recombination. Note typical wildtype ($nrl^{+/+}$) retina in right side of E" containing no evidence of cone lineage trace reporter in rod cells. **F.** With transgenic expression of ectopic Nrl in UV cones via *Tg[sws1:Nrl]*, many rod cells (4C12+) now co-localize the cone lineage tracer. Thus UV cones expressing Nrl transmute to become rod cells. Also shown: a lineage reporter-positive bipolar cell (long arrow). There were a total of 3 bipolar cells labeled in this section, assessed between the CMZ and ONH.

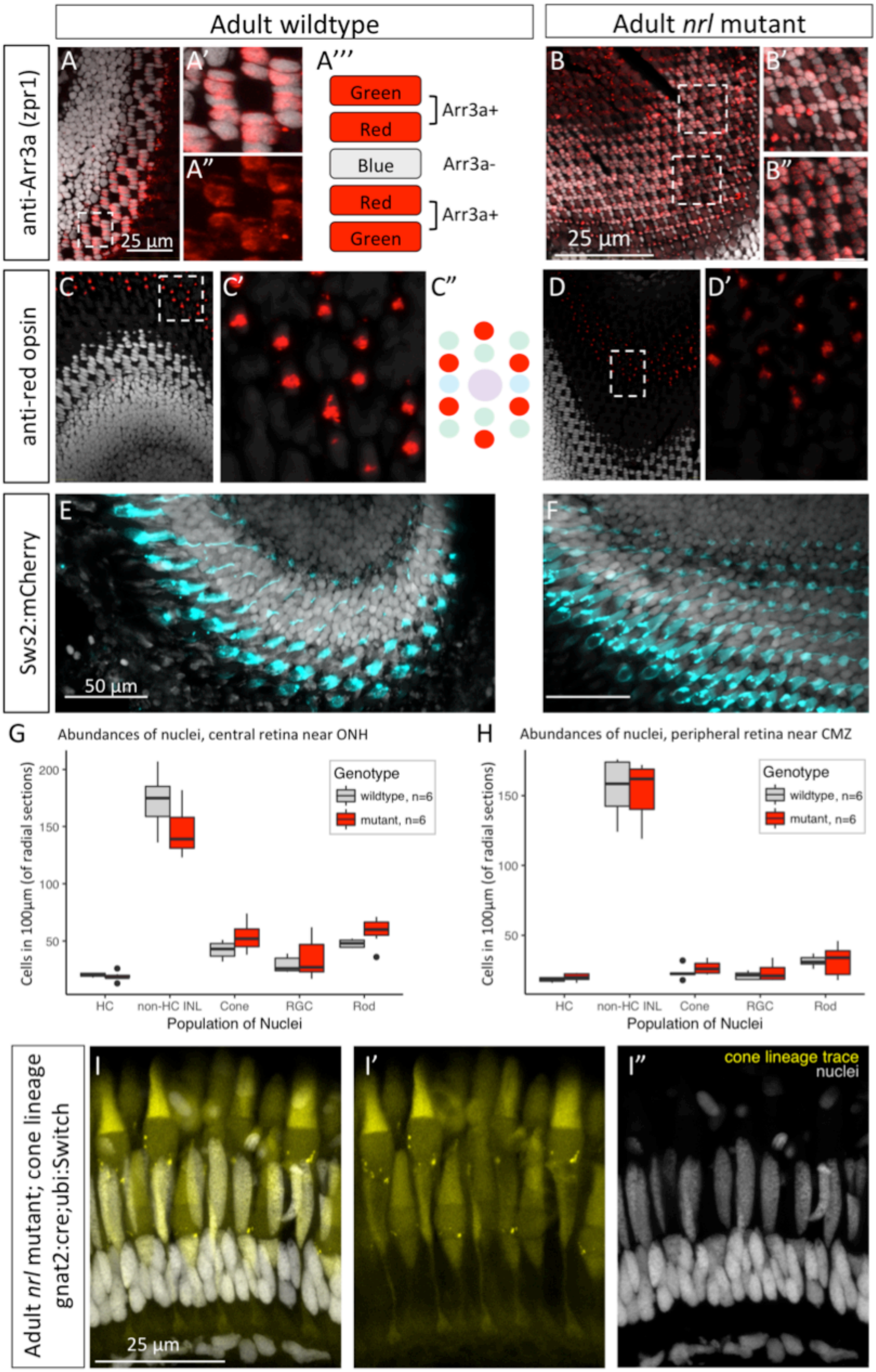

Figure S5. Adult retina of zebrafish *nrl*$^{-/-}$ mutants have no overt phenotypes…
(Legend on next page)

Figure S5. Adult retina of zebrafish *nrl*$^{-/-}$ mutants have no overt phenotypes regarding cell type abundances or cone sub-types. Relates to Figure 4 regarding lack of expected photoreceptor phenotypes in adult *nrl*$^{-/-}$ retina. **A-F.** Viewed in tangential (or somewhat oblique) cryosections, cone photoreceptors in adult zebrafish have a consistent and regularly repeated pattern (e.g. A" and C") that allows sensitive detection of phenotypes or discrepancies, and none are detected in *nrl*$^{-/-}$ mutant retina compared to wildtype. Sections are counterstained for cell nuclei (grey). Boxes outlined in main panels are included in adjacent insets (e.g. panel A has insets A', A", etc.). **A,B.** Double-cones (fused green- & red-sensitive cones) are detected as zpr1+ (an antibody against Arrestin3a) and display their typical repeating pattern, where each double cone flanks a zpr1- single cone (blue-sensitive cone). Double cones are zpr1+ and show typical inter-cellular organization/ positioning in *nrl*$^{-/-}$ mutant retina. **C,D.** Red-sensitive cone opsin (*lws*) immunolabelling reveals red cones in their expected pattern amongst other cone types (schematized in C") in both wildtype and *nrl*$^{-/-}$ mutant retinas. **E,F.** Blue-sensitive cones, detected with transgene *Tg[sws2:mCherry]* that drives mCherry under the *sws2* opsin promoter (pseudocoloured cyan), shows the expected rows – a repeating pattern and regular spacing of blue-sensitive cones – in *nrl*$^{-/-}$ mutant retina (F). **G,H.** Abundances of cell nuclei were determined in radial cryosections of adult retina (akin to sections in Fig. 3C & C') by counting DAPI+ nuclei in each retinal layer. Radial sections selected for analysis were cut parallel to the nasal-temporal axis of the eye and transected the optic nerve head. Counts of various retinal cell nuclei were made from a region of retina delineated by measuring 100 μm along the outer plexiform layer and then counting the cells in each layer that were below or above this line. Counts were made on the basis of a single 0.58μm thick optical section, captured from a 10μm thick cryosection. Cell types are morphologically distinct and/or determined by their location in retinal layers: horizontal cell nuclei, HC; cells of the Inner Nuclear Layer other than horizontal cells, non-HC INL; cone cell nuclei, Cone; retinal ganglion cells, RGC; rod cell nuclei, Rod. Panel (G) quantifies cells in a central region, adjacent to the optic nerve head, whereas Panel (H) assesses peripheral retina near the ciliary marginal zone. Wilcoxon rank sum tests of each population of neurons by genotype revealed no significant differences after Bonferroni correction for multiple comparisons. n=6 individuals of each genotype. **I.** Lineage tracing of cone photoreceptors (from *Tg[gnat2:Cre]* and ubi:Switch reporter as per Fig. S1A,B) in adult *nrl*$^{-/-}$ mutant retina shows normal abundance and morphology of cone cells. Further, there is no detectable signature of cone lineage tracing in the *nrl*$^{-/-}$ rod photoreceptor cells.

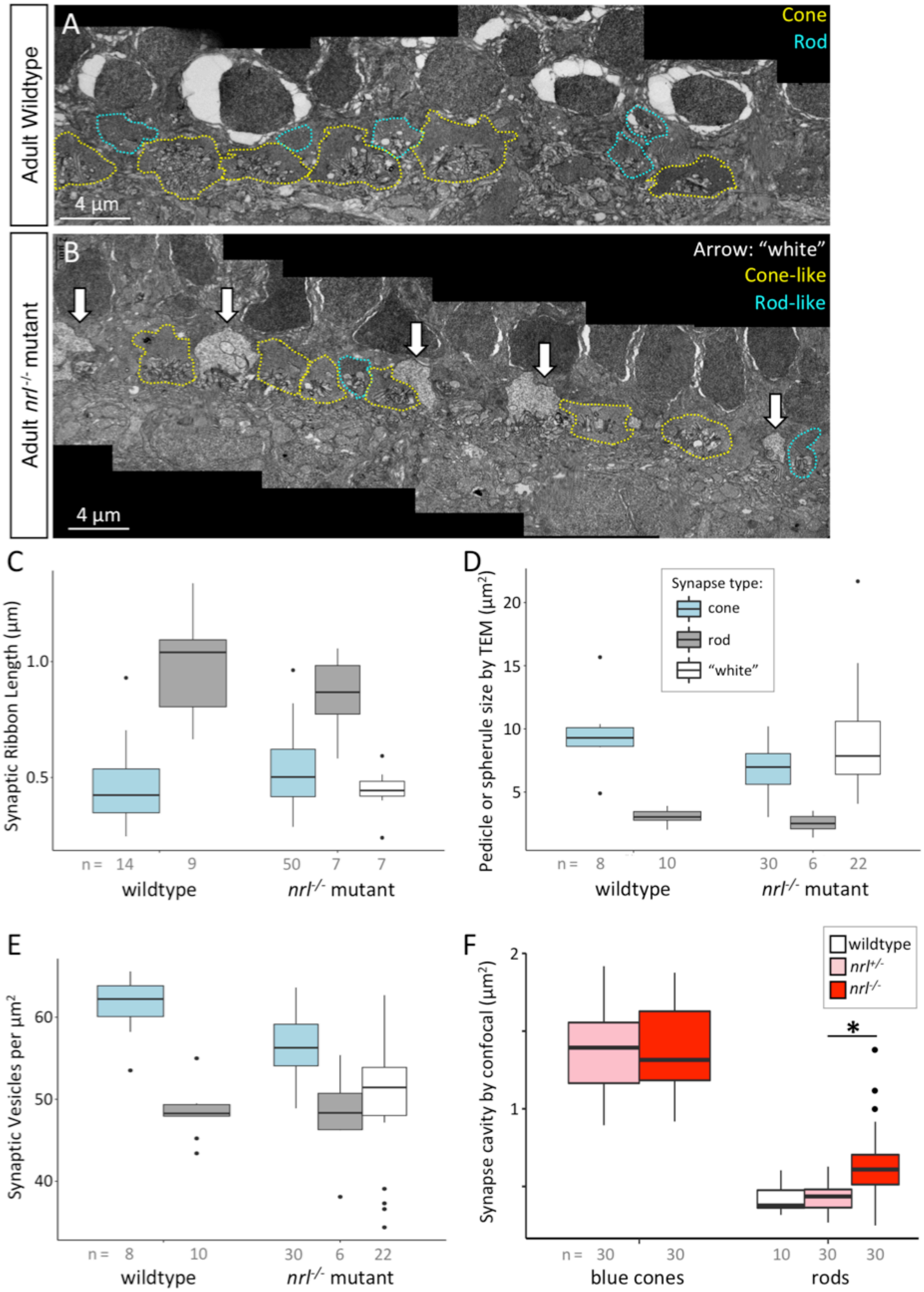

Figure S6. Photoreceptor synaptic terminals in adult retina of *nrl*$^{-/-}$ mutant zebrafish… (legend on next page)

Figure S6. Photoreceptor synaptic terminals in adult retina of *nrl*$^{-/-}$ mutant zebrafish suggest a requirement for Nrl in differentiation or maintenance of the rod synapse. Relates to Figure 6. **A,B.** Ultrastructure of Outer Plexiform Layer of adult retina, showing an expanded view of Fig. 6B,C. In wildtype adult zebrafish the photoreceptor synaptic terminals (panel A) include rod spherules (teal dotted lines) that are morphologically distinguishable from cone pedicles (yellow) based on their smaller size and smaller number of synaptic ribbons. In adult *nrl*$^{-/-}$ mutant retina, the photoreceptor terminals (panel B) are disrupted compared to wildtype: a paucity of rod spherules are recognizable despite the presence of a normal abundance of rod cells. Further, only in adult *nrl*$^{-/-}$ mutant outer nuclear layer, a subset of photoreceptor terminals were cone-like but extremely electon-lucent such that we denoted them ‘white synapses’ (white arrows). We suggest these white synapses may be *nrl*$^{-/-}$ rod synapses because they are only present in mutants, and they would account for the disparity of only observing a paucity of rod spherules despite the large abundance of rod cells. Alternatively, the white synapses might be disrupted cone pedicles, but the data in panel (F) below is more consistent with *nrl*$^{-/-}$ rod synapses being present and somewhat cone-like. **C-E.** Ultrastructural features of photoreceptor synaptic terminals were quantified in rod spherules, cone pedicles and ‘white synpases’ in each genotype. Rod and cones showed no striking difference based on genotype. White synapses are somewhat more cone-like (compared to a typical rod spherule) with respect to synaptic ribbon length (C), photoreceptor terminal size (D), and somewhat more variable with respect to density of synaptic vesicles (E). n= number of synaptic terminals, where wildtype data is from synapses imaged across two animals; mutant data from synapses imaged across 5 animals. Floating points are statistical outliers. **F.** Rod spherules were characterized in sibling vs. *nrl*$^{-/-}$ mutant *Tg[rh1:gfp]* adult zebrafish by measuring the area of the synaptic cavity (the synaptic cleft contained between arms of GFP+ rod spherule) in confocal images of radial sections (e.g. Fig. 4D, D’). Similarly, the synaptic cavity of blue cone pedicles were quantified in fish bearing *Tg[sws2:mCherry]*. Blue cone pedicles were not apparently different between genotypes. Rod spherules had significantly larger synaptic cavities (and thus were somewhat more cone-like) in *nrl*$^{-/-}$ rods ($p = 4.319e\text{-}08$, $n = 10$ synapses per fish, 3 fish per genotype for mutants and heterozygotes, and 1 wildtype fish), under Mann Whitney U comparison. Floating points are statistical outliers.

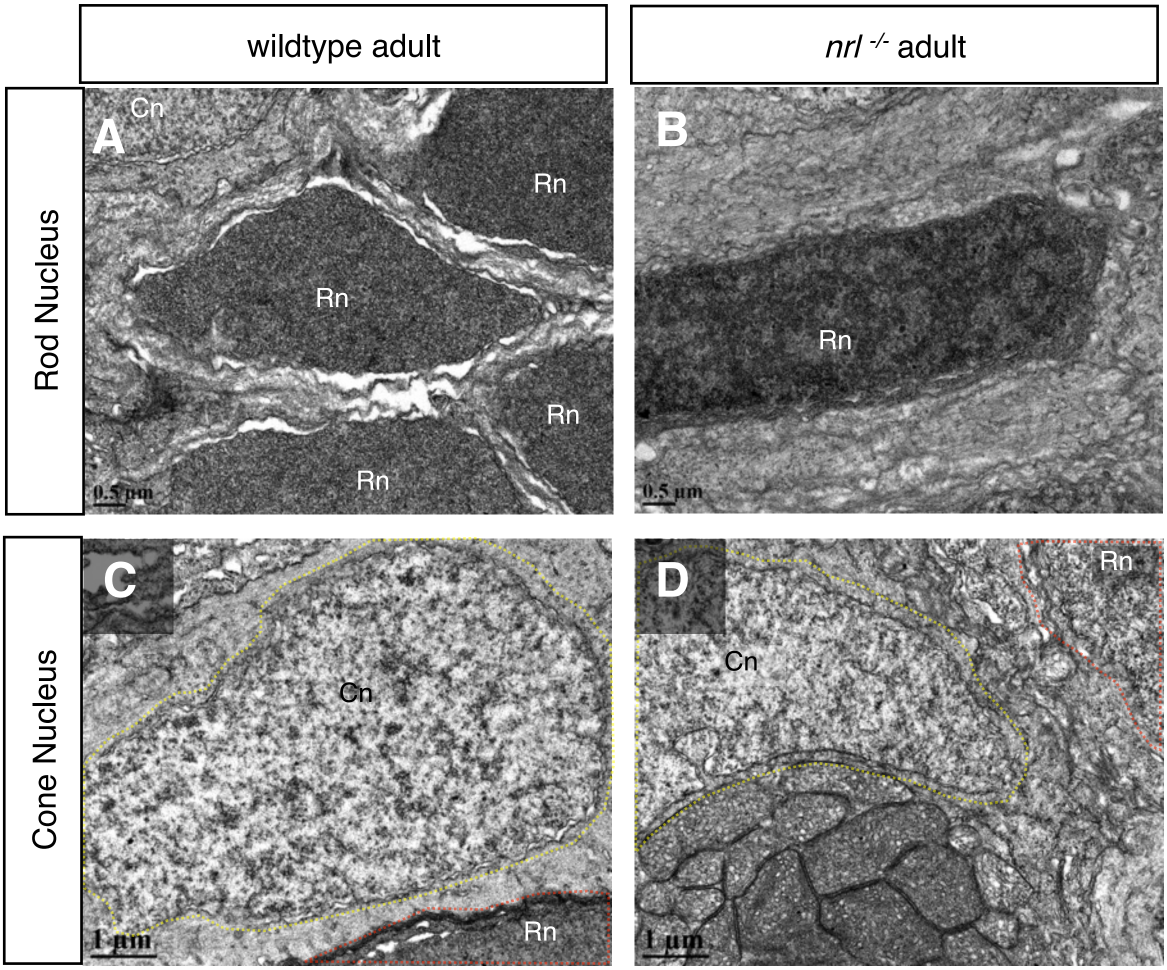

Figure S7. Photoreceptor nuclei in adult retina of *nrl*$^{-/-}$ mutant zebrafish. Relates to Figure 6. In *nrl*$^{-/-}$ adult retina the cone nuclei appear normal, but rod nuclei look somewhat 'cone-like', compared to wildtype, in that they have less homogeneous electron density.

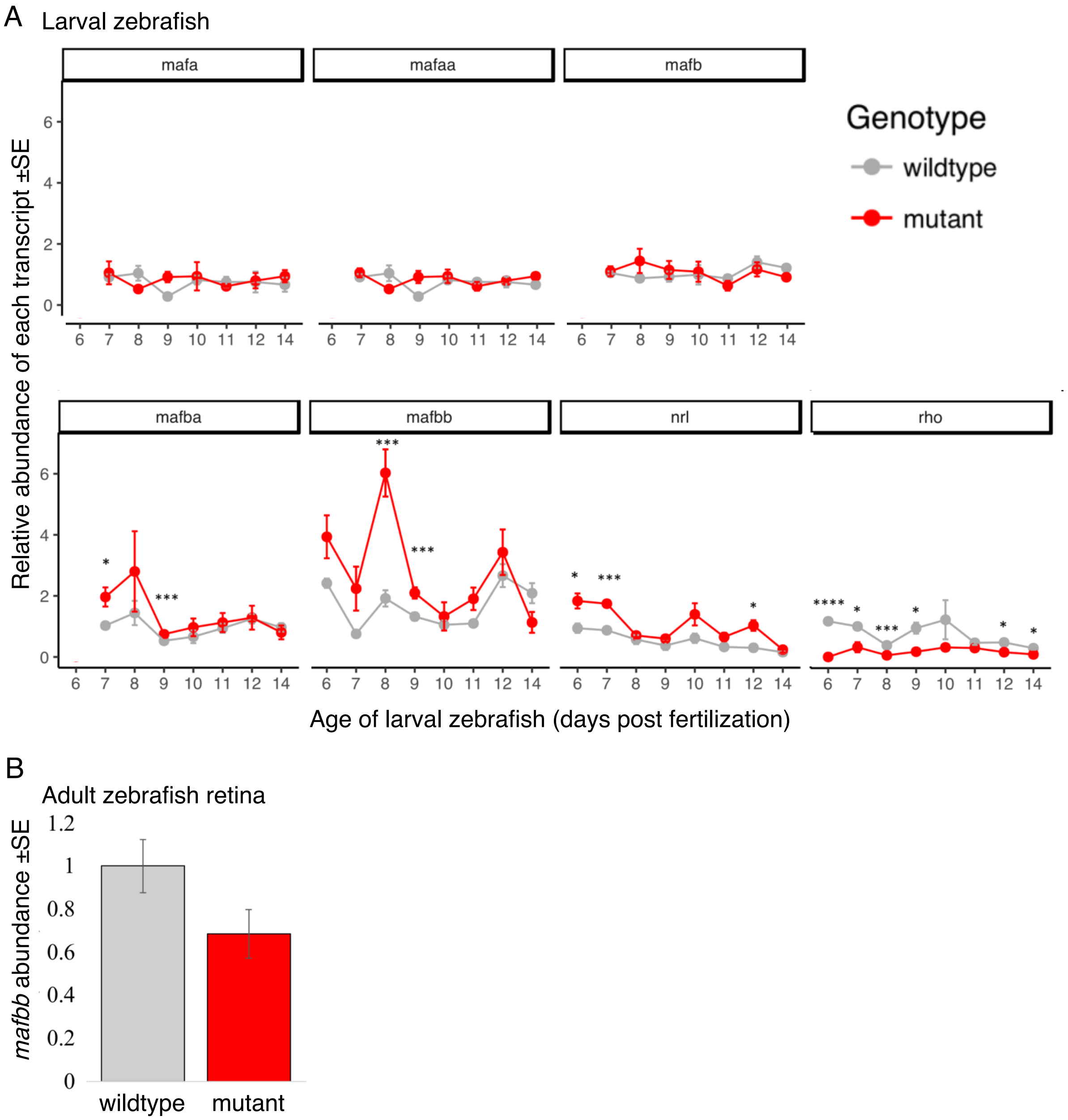

Figure S8. Relative abundances of various Maf family transcripts is dynamic over development, including over the times when rods begin to appear in *nrl*$^{-/-}$ mutants. Relates to Figure 5. The transcript abundances rarely vary between genotypes; One exception was a brief spike in relative abundance of *mafbb* midway thru larval development. **A.** transcript abundance detected in whole larvae at various developmental timepoints. Biological replicates including five larvae each and three biological replicates were averaged per timepoint. *p<0.05; p<0.01; p<0.001. **B. *mafbb* transcripts abundance was not increased in adult neural retina.** Because *mafbb* was the only gene that seemed likely to vary by genotype during larval development, we assessed its relative abundance in adult *nrl*$^{-/-}$ retina but did not find that it was increased in a manner that might inform how *nrl*$^{-/-}$ rod cells are specified. n=5 per genotype, p=0.168.

TRANSPARENT METHODS

Animal Ethics

All protocols involving zebrafish husbandry or experimentation were approved by institutional ethics Committees at the University of Alberta (protocol # AUP00000077), as overseen by the Canadian Council on Animal Care.

Transgene Construct Cloning

Transgene expression plasmids were created using by incorporating gBlock Gene Fragments (from IDT, Integrated DNA Technologies, Coralville, Iowa) into the Multisite Gateway Cloning system. Zebrafish and mouse *nrl*, (appended with attB1 and attB2r flanking sequences, including a Kozak sequence, and a N-terminal 3xFLAG tag on Mouse Nrl), were both ordered as a gBlock Gene Fragment. Where applicable, silent mutations were engineered into the CDS of the various peptides in order to optimize for zebrafish codon bias and to circumvent nucleotide composition/ complexity requirements; in all cases, the predicted peptide produced is wholly wildtype aside from the relevant N-terminal tag. Received gBlocks were then processed for Multisite Gateway Cloning using the zebrafish Tol2Kit reagents (Kwan et al., 2007) and Gateway system (Life Technologies), and were ultimately recombined with regulatory sequences of p5E-sws1 (Fraser et al., 2013) and a p3E-polyA sequence into an expression vector. The expression vector backbone pDestTol2CG2 has a cmlc:eGFP reporter that drives eGFP expression in the heart muscle cells, to aid in identifying transgenic fish. Transgene expression constructs driving Cre recombinase expression were similarly assembled and cloned as above in front of p5E-gnat2 promoter (Suzuki et al., 2013, Kennedy et al., 2007); The p5E-gnat2 gateway plasmid was a kind gift from Dr. Rachel Wong of the University of Washington, USA.

Morpholino and Plasmid Injections

Morpholino was injected into single cell embryos bearing rh1:eGFP and either sws1:nfsb-mCherry or sws2:nfsb-mCherry were injected with 10ng of standard control (CCT CTT ACC TCA GTT ACA ATT TAT A) or *nrl* splice-blocking morpholino (ACG TGT CAG ATC ATA CCT GTG AAG T) (Genetools, LLC, Philomath, Oregon), delivered to the yolk as a 5nL bolus. Morpholinos were first suspended in water and then diluted into 0.1M potassium chloride with 0.1% phenol red added to assess injection success. Morphants were reared to 4 dpf and then processed for retinal mounting and imaged.

Transgene construct injection mixtures were prepared by diluting 750ng plasmid construct and 250ng Tol2 mRNA into 0.1M potassium chloride with 0.1% phenol red to a total volume of 10 μL. These solutions (5-10nL) were delivered to the yolk of the single cell embryo. Injected animals were raised to 2 dpf, and then inspected for eGFP-expressing heart cells, the marker of pDestTol2CG2 plasmid presence. Larvae with GFP-expressing heart cells were reared to adulthood, and germline transmission of transgenes identified in the subsequent generation.

CRISPR Mutagenesis

To engineer allele *nrl*^{ua5014}, a mixture of three guide RNAs (gRNA) targeting the first coding exon of zebrafish nrl were delivered. These gRNA were designed in Geneious

R9.1.7 and synthesized per Gagnon and colleagues (Gagnon et al., 2014), but using mMessageMachine SP6 kit (Invitrogen). The gRNAs were mixed with protein Cas9 (NEB), allowed 5 minutes at 37°C to assemble into ribonucleoprotein complexes, and the mixture was microinjected into the cell of zebrafish embryos at the 1 cell-stage. Animals at 2 dpf were sacrificed for high-resolution meltcurve (HRM) analysis of *nrl* cutting (STAR methods for primers; methods as previously described (Fleisch et al., 2013, Pillay et al., 2013).
For *nrl*^{ua5009}, the 5'-most guide RNA used above, and a second guide RNA targeting eGFP (CR.GFP.GA5'.break, STAR methods) were mixed together and allowed to complex with protein Cas9. This mixture was microinjected into the cell of 1 cell-stage zebrafish embryos, bearing the ubi:eGFP transgene (STAR Methods), which promotes eGFP expression in all cells. At 2 dpf, a subset of animals with disrupted eGFP were checked for nrl mutation, and the remainder of eGFP-disrupted fish were reared to adulthood and visually screened for germline transmission of mutant eGFP (carriers had no eGFP fluorescence, but the transgene bears a cardiac-expressed RFP marker cassette (Fraser et al., 2013)). Fish with germline CRISPR editing were then checked for germline-transmitted nrl mutation, the *nrl*^{ua5009} allele was recovered, and ubi:eGFP^{mutant} was bred out in subsequent generations.

Genotyping RFLP

Genotyping the *nrl*^{ua5009} allele leveraged a *TaqI* restriction enzyme cut site unique to the mutant allele. PCR amplification of *nrl*, using primers listed in table, was performed from genomic DNA. PCR parameters: 96°C 2min, 35x [56°C 15s, 72°C 1min, 96°C 15s], 72°C 5 minutes. PCR products were digested with *TaqI* restriction enzyme and analyzed by gel electrophoresis; the ~675bp amplicon from the *nrl*^{ua5009} allele is cloven into two products. The *nrl*^{ua5014} allele was genotyped using these same PCR conditions and then resolving its larger amplicon, caused by the 109bp CRISPR-induced insert, via gel electrophoresis.

5'RACE to characterize mutant *nrl* transcript

RNA was purified from adult zebrafish retina or pools of 4 dpf larvae using an RNeasy Minikit (Qiagen catalogue # 74104. Hilden, Germany). RNA sample quality was determined by Bioanalyzer 6000 RNA nano chip (Agilent catalogue # 5067-1512. Santa Clara, CA, USA), selecting for samples with RIN numbers >9. 5' RACE was performed using a Clontech SMARTer RACE 5'/3' kit (Takarabio catalogue # 634858. Kusatsu, Shiga Prefecture, Japan) with a primer in the third coding exon of *nrl* transcript ENSDART00000168271.3 (GATTACGCCAAGCTTTCTGTTCAGCTCGCGCACAGACAGACTC). Products were characterized on a 1% agarose gel and then purified using a Nucleospin Gel and PCR cleanup kit (Machery-Nagel # 740609.10. Düren, Germany). In fusion Cloning was performed using the Clontech SMARTer RACE 5'/3' kit and identity of the 5'RACE products were verified, including appropriate presence/absence of the engineered mutation in allele ua5009, by sequencing using standard M13 primers.

RNA Isolation and Quantitative real time polymerase chain reaction (qRT-PCR)

Neural retinae were dissected from adult zebrafish dark adapted for 12 hours between 5:30 PM and 6:30 PM and stored in RNAlater (Ambion) at 4°C until extraction. Total RNA was isolated from one retina per fish using RNeasy Lipid/Tissue Mini Kit (Qiagen)

according to the manufacturer's instructions. Samples were homogenized in 700 μL of Qiazol (Qiagen) containing 1% of β-mercaptoethanol (Sigma) with a rotor stator homogenizer (VMR) and put through an "on column" DNAse digestion using DNAse I (Qiagen). RNA concentration was determined by a Nanodrop spectrophotometer (GE Healthcare 28 9244-02) and Agilent 2100 Bioanalyzer (Agilent RNA 6000 NanoChip). For each sample, 100ng of total RNA was reverse transcribed using qScript cDNA Supermix (Quanta Biosciences) as per manufacturer's instructions. cDNA was diluted 1:10 in Nuclease-free H_2O (Ambion) and stored at -20°C until use. Primers were designed using the Primer 3 algorithm in Geneious R.9.1.7 (Kearse et al., 2012)) to amplify cDNA products. All primers were validated using a standard serial dilution to determine efficiency under MIQE guidelines (Bustin *et al.*, 2009). Dissociation curves were analyzed in 7500 Software v1.4.1 (Applied Biosystems, 2011) and only single products were detected. *β-actin* was used as endogenous control gene for normalization (Tang *et al.*, 2007; Fleisch *et al.*, 2013); we found no evidence that *β-actin* transcript abundance varied between genotypes (e.g. relative abundance of *β-actin* normalized to *gapdh* abundance was 100±1.3% in wildtype and 99±0.64% in *nrl*$^{-/-}$ mutants, n=3). Primer sequences can be found in STAR Methods. Each qRT-PCR reaction consisted of 2.5 µL of 3.2 µM primer solution, 5 µL of 2x (*Dynamite*) qPCR MasterMix (MBSU, University of Alberta) and 2.5 µL of cDNA in a 10 µL reaction volume. Technical replicates were performed in triplicate. RT-qPCR reactions were run on 7500 Fast Mode (pre-incubation 95°C, 2:00 min; 2 step amplification 95 °C 15s, 60 °C 1:00 min; 40 cycles; dissociation 95°C 15s, 60°C 20s, 95°C 15s, 60°C 15s) using the 7500 Fast Real-Time PCR System (Applied Biosystems). Biological samples (n= 5-6) each represent retinas from independent fish.

<u>Western Blots using custom anti-Nrl antibody</u>

Adult zebrafish neural retinas were dissected and total protein was extracted by homogenizing samples in a standard lysis buffer (4mL 0.5M Hepes, 20mL 100% glycerol, 10mL 5M NaCl, 0.039g $MgCl_2$, 40uL 0.5M EDTA, 100uL Triton X-100, 65.86mL ddH_2O) with 1:200 protease inhibitor cocktail (EMD Millipore/VWR catalogue #CA80053-852, Darmstadt, Germany) with a rotor stator homogenizer (VWR catalogue #47747-370, Radnor, PA, USA). Homogenate was centrifuged at 13000 rpm for 8 minutes and supernatant was collected. Total protein in the supernatant was quantified using a Qubit Fluorometer (Invitrogen calatogue #Q32857, Carlsbad, CA USA). 30ug of protein was diluted with 2X SDS loading dye (12.5 mM Tris, 2% glycerol, 0.4% SDS, 0.2% β-mercaptoethanol, 0.1% bromophenol blue), and loaded into a 10% acrylamide stacking gel, followed by a 25% acrylamide separating gel. Protein was then transferred to a nitrocellulose membrane and blocked with 5% skim milk in tris buffered saline solution with 0.1% Tween (TBST). Membranes were probed with a custom polyclonal antibody raised against zebrafish Nrl, diluted 1:2000 in TBS. Antigen for the custom anti-Nrl antibody (commissioned from Genscript, Piscataway, NJ, USA) was recombinant protein matching the C-terminal 112 amino acids of zebrafish Nrl (residues 301-412 of GenBank: AAI63220.1). Antibody was affinity purified from the serum of a rabbit host. The blots probed with α-Nrl antibody were detected using 1:5000 goat α-rabbit HRP secondary antibody (Jackson Immunoresearch catalogue #111-005-003, West Grove, PA, USA) in TBST with 1% milk. Blots were developed using SuperSignal West Fempto Chemiluminescent substrate (Thermo Fischer scienctific catalogue #34095. Waltham, MA,

USA) and visualized using ChemiDoc MP Imaging System (Hercules, CA, USA. Catalogue #17001402). The blot was then stripped and re-probed with a 1:5000 dilution of anti-β-Actin antibody (Sigma catalogue #A2066, St. Louis, MO, USA) in TBST with 1% milk. The anti-β-Actin antibody was detected using a 1:10000 dilution of goat α-mouse HRP secondary antibody (Jackson Immunoresearch catalogue #115-035-003, West Grove, PA, USA). The intensity of the bands was then analyzed using ImageJ (National Institutes of Health, Bethesda, MD, USA), to calculate the ratio of the intensity of the Nrl immunoreactivity compared to the intensity of the β-actin bands.

Wholemount immunostaining

Anaesthetized larvae were fixed with 4% paraformaldehyde in 0.1M phosphate buffer with 5% sucrose pH 7.4; fixation was at room temperature for at least two hours, or overnight at 4°C. Fixed larvae were washed from PFA with phosphate buffered saline pH 7.4 with 0.1% Tween20 ("PBSTw"), then prepared for immunohistochemistry. Fish were washed in pure water 5 minutes, -20°C acetone for 7 minutes, rinsed out of acetone with PBSTw + 1.0% DMSO, and then blocked for at least 30 minutes in 10% normal goat serum (ThermoFisher) in PBSTw. Blocking solution was drained away, and primary antibody was applied. Antibody incubations were mixed 1:100 for 10C9.1 or 1:500 for other primary antibodies, and 1:1000 for secondary antibodies, and incubated at 4°C at least overnight. Larvae were washed from antibody over 2x5min and then 2x1hr washes in PBSTw, and then transferred to 70% glycerol in PBSTw until equilibrated. The retinas of equilibrated larvae were then prepared for mounting.

Wholemount larval retinal dissections

For mounting whole retinas, glycerol-equilibrated larvae were relieved of their lenses using microscalpels (electrolytically-sharpened tungsten wire needles) as previously described (Conrad et al., 1993). After de-lensing, the corneal and scleral tissues covering the retina were cut and folded back, and the retina removed from the socket with the tungsten needles using a scooping manoeuvre. The retinas were then positioned vitreal-side down upon slides, and cover slips positioned upon the scleral side. Excess 70% glycerol was added as a mounting medium, and the slides were then imaged as described below.

Cryosectioning and immunocytochemistry

Larvae were fixed as described above and, after PFA was washed away, subjected to a graded series of sucrose washes, from 5% to 20% sucrose in 0.1M phosphate buffer, and cryoprotected overnight in the final 20% sucrose stage. After, the fixed larvae were transferred to a 2:1 mixture of 20% sucrose buffer : OCT cryosection fluid (TissueTek) for one hour, then transferred to a final 1:1 mixture of the same. After equilibrating to this new buffer, fixed larvae were embedded into plastic molds, frozen at -80ºC for at least two hours, and then sectioned at 10 um thickness. Cut sections were allowed to air dry 30 minutes before storage at -80ºC at least overnight. Retrieved sections were warmed to room temperature over 20 minutes, then washed 3x5min in PBS + 0.1% Tween20 ("PBSTw") to remove sectioning media residue. Sections were then blocked and stained as for wholemount immunohistochemistry (above), then covered with 70% glycerol in

PBSTw as a mounting medium, mounted under a coverslip, and imaged as previously described (DuVal et al., 2014).

in situ hybridization on retinal cryosections

In situ hybridization on frozen sections was performed as previously described (Raymond et al., 1993). Briefly, frozen sections were thawed and rehydrated, then immediately re-fixed in 4% PFA to help tissue adhere to slides. Sectioned tissue was digested briefly with proteinase K and then re-fixed with 4% PFA. Sections were then acetylated with a mixture of triethanolamine and acetic anhydride, then dehydrated in a graded ethanol series with diluted 2x sodium citrate buffer. Tissues were pre-hybridized in Hauptmann's buffer, then hybridized with riboprobe overnight at 70°C using 1μg/mL DIG-labeled riboprobe. Probe was washed off in a graded series of sodium citrate buffer diluted in maleate buffer as previously described. Sections were blocked and then incubated with anti-DIG-conjugated alkaline phosphatase antibody (Roche) at 1:5000 dilution overnight. Alkaline phosphatase chromogen reaction was performed, terminated with excess alkaline phosphate buffer and subsequent fixation with 4% PFA. Sections were mounted with glycerol. Developed sections were imaged on an Axioscope A.1 microscope (Carl Zeiss MicroImaging, Oberkochen) with 12 bit MacroFIRE camera (Optronics, Goleta, CA, USA).

Riboprobes to detect *rh1, sws1* and *rh2* opsins were produced as per our previous methods (Allison et al., 2010). Riboprobes against *rh2* opsin a cocktail including DIG-labelled riboprobes against all of *rh2-1*, *rh2-1*, and *rh2-3*, akin to our previous methods (Allison et al., 2010).

Riboprobes to detect *nrl* and *nr2e3* were produced using primers listed in Table S1, cloned into pCS2+ or used directly as template to produce riboprobes of 972 and 867 bp, respectively.

Confocal and stereomicroscopy

Confocal microscopy was performed with an LSM 700 confocal microscope mounted on a Zeiss Axio observer. Images were acquired with ZEN 2010 (v6.0, Carl Zeiss AG, Oberkochen, Germany). Micrographs were taken using 63x oil immersion (numerical aperture of 1.4) and 20x objectives (numerical aperture of 0.8). Images acquired with the confocal microscope were captured with gain adjusted to avoid any empty or saturated pixels, and after acquisition, image minima, maxima, and gamma were adjusted in Fiji (ImageJ, Version 2.0.0-rc-54/1.51h, NIH, Bethesda, MD, USA) to improve contrast. Stereoscopy images were taken as previously described (Duval et al., 2013) using brightfield and fluorescent channels in separate photos. Where relevant, brightfield images were converted to grayscale and merged in Fiji with fluorescent channel images to improve GFP visibility.

Transmission Electron Microscopy

Adult eyes with the lenses removed were fixed overnight at 4ºC. Fixative was 2.5% glutaraldehyde, 2% paraformaldehyde in 0.1M phosphate buffer PH 7.4. Fixative was washed out of the samples, which were then subjected to a graded series of ethanol washes, and then infiltrated with resin and embedded. Samples were then sectioned on a Richert-Jung Ultracut E Ultramicrotome to sections of 70-90 nm thickness. Gridded sections were then stained with uranyl acetate and lead citrate, and imaged at 80 kV on a

FEI COMPANY transmission electron microscope, model Morgagni 268 (FEI company, Hillsboro, Oregon). Images acquired with a Gatan Orius CCD camera using Gatan DigitalMicrograph image acquisition software, version 1.81.78 (Gatan, Inc., Pleasanton, CA).

Wholemount larvae photoreceptor quantification

In larval whole mounted eyes, the dorsal-ventral axis is readily apparent while imaging. After confocal imaging, using Fiji image analysis software a 100x100 μm boundary was positioned just dorsal to and centered above the optic nerve head of the retina. Within the delineated box, the *Process>Smooth* function of Fiji tamed wild background pixels, and then the *Process>Find Maxima* function was used to efficiently count fluorescently labeled photoreceptors.

Quantification of adult nuclei in histological sections

To compare the relative abundance of retinal nuclei between adult wildtype and *nrl*$^{-/-}$ mutant zebrafish, a counting region of interest (ROI) boundary was first established, consisting of 100μm measured along the outer plexiform layer in the indicated region. From this, an ROI boundary was drawn to encapsulate all the nuclei within this stretch, taking into account the bends in the retinal tissue. All nuclei were hand-counted except for the non-HC INL nuclei, which were counted as for wholemount larval photoreceptors (above).

Ultrastructural synapse and chromatin appearance quantification

For all quantification and analysis, transmission electron micrographs of synapses and nuclei were imported into Fiji, and analysed as described. To quantify synaptic vesicle density, a region of interest (ROI) boundary was drawn around a photoreceptor synapse that excluded bipolar and horizontal cell processes. The interior of the ROI was then processed in the following way in Fiji: First, *Process>Subtract Background*, rolling ball radius = 5.0 pixels, “light background”. Next, background pixel noise was suppressed using *Process>Filter>Median*, radius = 1.0 pixel. The image was then thresholded with *Image>Adjust>Threshold*, auto. Finally, *Analyze>Analyze Particles* was used to count particles, with minimum and maximum particle radius set to 0.001 and 0.005μm (based on previous calibration). The area of the total ROI was recorded as the area of the synapse.

To measure synaptic ribbon length, only ribbons meeting particular criteria were included: only ribbons with a clear synaptic ribbon “head” were measured; numerous synapses with oblique cut angles showed shadows of synapse ribbons slightly out of plane with the image, and the lengths of these shadows varied considerably (data not shown). Thus, only synaptic ribbons cut *en face* were measured. The segmented line tool of Fiji was used to trace the length of each ribbon, calibrated to the scale bar in each micrograph.

Statistical Analysis

Statistical analyses (described in associated Figure legends, including Mann-Whitney-U, Wilcoxon ranked sum tests) and plot generation for larval photoreceptor abundances, synaptic vesicle density and ribbon length, relative abundances of nuclei in retinal

sections, and rod nuclear sizes were performed in R (version 3.4.1, R Foundation for Statistical Computing, Vienna, Austria).

All RT-qPCR data is presented as mean ± Standard Error of Mean (SEM) and is standardized to wild type (AB strain) transcript abundances. To analyze differences in mean expression between wild type and mutant larvae and retinal tissue, a Mann-Whitney Test was performed using GraphPad Prism (Version 7.02 for Windows, or 8.4.2 for Mac, GraphPad Software, La Jolla California USA, www.graphpad.com). Two outlier data points were removed, after their identification with both ROUT analysis (Q = 5%) (α=0.05) and Grubb's test (α=0.05) in GraphPad Prism; the same two outliers were identified when was implemented (Q = 5%, α=0.05). Statistical significances are denoted in each respective Figure legend.

Supplemental Table S1 – Key Resources

REAGENT or RESOURCE	SOURCE	IDENTIFIER
Antibodies		
Rat anti-trout UV cone opsin; 10C9.1	(DuVal et al., 2014)	10C9.1; ZFIN ID: ZDB-ATB-140728-2
Mouse anti-bovine rhodopsin; 4C12	(Morris et al., 2005)	4C12; ZFIN ID: ZDB-ATB-090506-2
Rabbit anti-GFP	Invitrogen	Cat#A11122
Rabbit anti-FLAG tag	ThermoFisher	Cat#PA1-984B
Rabbit Genscript: anti zebrafish NRL	This paper	N/A
Rabbit anti-HA tag	Abcam	Cat#ab137838
Mouse anti-Arr3a; zpr-1	ZIRC	Cat# Zpr-1<br>ZFin ID: ZDB-ATB-081002-43
Alexa Fluor 647 donkey anti-rat	Invitrogen	Cat#A21472
Alexa Fluor 647 donkey anti-mouse	Invitrogen	Cat#A31571
Alexa Fluor 555 donkey anti-mouse	Invitrogen	Cat#A31570
Alexa Fluor 488 donkey anti-mouse	Invitrogen	Cat#A21202
Biological Samples		
Gateway-compatible middle element donor (empty) vector: pDONR221	Tol2Kit; http://tol2kit.genetics.utah.edu/index.php/Main_Page	pDONR221
Multi-site gateway-compatible destination (empty) vector: pDestTol2CG2	Tol2Kit; http://tol2kit.genetics.utah.edu/index.php/PDestTol2CG2	pDestTol2CG2
Gateway-compatible middle element (“pME”) nfsb-mCherry	(Davison et al., 2007)	N/A
Gateway-compatible 5’ element (“p5E”) ubi:loxP-eGFP-loxP	Addgene; (Mosimann et al., 2011)	Addgene#27322
Gateway-compatible 3’ element (polyadenylation signal) vector: p3E-polyA	Tol2Kit; http://tol2kit.genetics.utah.edu/index.php/P3E-polyA	P3E-polyA
Gateway-compatible 5’ element (*sws1* regulatory sequences) vector: p5E-sws1	(Takechi et al., 2003)	P5E-sws1
Gateway-compatible 5’ element (*gnat2* regulatory sequences) vector: p5E-gnat2	(Suzuki et al., 2013)	P5E-gnat2

Chemicals, Peptides, and Recombinant Proteins

RNase-Free DNase I Set	Qiagen	Cat#79254
2x (*Dynamite*) qPCR MasterMix	Molecular Biology Service Unit (MBSU), University of Alberta	N/A
qScript cDNA SuperMix	Quanta Biosciences	Cat#95048-100
RNAlater RNA Stabilization Solution	Ambion	Cat#AM7020
β-mercaptoethanol	Sigma	Cat#M3148
Nuclease-free H2O	Ambion	Cat#4387936
Critical Commercial Assays		
RNeasy Mini Kit	Qiagen	Cat#74104
RNeasy Lipid Tissue Mini Kit	Qiagen	Cat#74804

Experimental Models: Organisms/Strains		
Zebrafish: tg[ubi:lox-GFP-lox-nfsb-mCherry; cmlc:RFP]ua3140: ua3140	This paper	N/A
Zebrafish: tg[sws1:nfsb-mCherry]q28tg : sws1:nfsb-mCh	Yoshimatsu et al. 2016 (Yoshimatsu et al., 2016)	ZDB-ALT-160425-1
Zebrafish: tg[sws2:nfsb-mCherry]q30tg : sws2:nfsb-mCh	D'Orazi et al., 2016 (D'Orazi et al., 2016)	ZDB-ALT-160425-3
Zebrafish: tg[rh1:GFP]kj2 : rh1:GFP	Hamaoka et al, 2002. (Hamaoka et al., 2002)	ZDB-ALT-060830-4
Zebrafish: Tg(sws1:GFP)^{kj9} : sws1:GFP	Takechi, Hamaoka, and Kawamura, 2003. (Takechi et al., 2003)	ZDB-ALT-080227-1
Zebrafish: tg[sws1:zebrafish nr]ua3162 : sws1:nrl	This paper	N/A
Zebrafish: tg[gnat2:cre]ua3162 : gnat2:cre	This paper	N/A
Zebrafish: Tg(ubb:lox2272-loxP-RFP-lox2272-CFP-loxP-YFP)a131 : zebrabow	Pan et al., 2013 (Pan et al., 2013)	ZDB-ALT-130816-2
Zebrafish: tg[sws1:FLAG-mouseNRL]ua3174 : sws1:mouseNRL	This paper	N/A
Oligonucleotides		
Primer: nrl genotyping Forward TGAACAACAGCTTCCAGCGAT	This paper	20160516.NCBI.nrl .E1F
Primer: nrl genotyping Reverse AGCTGTAAAACTTTGCATTCACG	This paper	20160516.NCBI.nrl .E1R
Primer: nrl High Resolution Meltcurve analysis Forward GTAAAGCCTGACACCCCTCC	This paper	N/A
Primer: nrl HRM Reverse CTGGAGCTCAGGCTGGAGT	This paper	N/A

Primer: nrl qPCR Forward AGCCTTCGCATCCCAACA	This paper	N/A
Primer: nrl qPCR Reverse CAGTGTCCGTTCAAGTGTGTCA	This paper	N/A
Primer: nr2e3 qPCR Forward CAACTGCCCTCTGCTGTCTCT	This paper	N/A
Primer: nr2e3 qPCR Reverse CTTGCAGAACCCTCACATCTGA	This paper	N/A
Primer: rhodopsin qPCR Forward CCCTGCCCGCCTTCTT	This paper	N/A
Primer: rhodopsin qPCR Reverse CGGAACTGCTTGTTCATGCA	This paper	N/A
Primer: sws1 qPCR Forward TCCTCCCGCAGCACATTTAC	This paper	N/A
Primer: sws1 qPCR Reverse AAAGTTACGGGATTTGAACAATCAG	This paper	N/A
Primer: mafa qPCR Forward CGAGGAGTCACCAAAGTGTTA	This paper	N/A
Primer: mafa qPCR Reverse ATCCGTGGCACAGTCTATTG	This paper	N/A
Primer: mafaa qPCR Forward TGAACTGAGGTTGAGGGGACTTG	This paper	N/A
Primer: mafaa qPCR Reverse ATGTCCCGCATGCAGGATTG	This paper	N/A
Primer: mafb qPCR Forward CGACGCGTACAAGGAGAAATA	This paper	N/A
Primer: mafb qPCR Reverse AAGAAGTGGCGAGCAGAAA	This paper	N/A
Primer: mafba qPCR Forward GAGAGACGCCTACAAACTCAAA	This paper	N/A
Primer: mafba qPCR Reverse ACACGCACTCACATGAAGAA	This paper	N/A
Primer: mafbb qPCR Forward TTGATCGAAACATCAGCAGAAATC	This paper	N/A
Primer: mafbb qPCR Reverse TCATGTCAAAGTCACCGTAGTC	This paper	N/A
Primer: gapdh qPCR Forward CCACCCCCAATGTCTCTGTT	This paper	N/A
Primer: gapdh qPCR Reverse TCATACTTGGCAGGTTTCTCAA	This paper	N/A
Primer: B-actin qPCR Forward CGGACAGGTCATCACCATTG	(Fleisch et al., 2013)	N/A
Primer: B-actin qPCR Reverse GATGTCGACGTCACACTTCA	(Fleisch et al., 2013)	N/A

Primer: nrl in situ probe template amplicon Forward<br>(adds HindII digest site for plasmid insertion to pCS2+)<br>AAGCTTCGTGCGCCTTGAAAACTGAA	This paper	'160711.NCBI.ISHnrl.F
Primer: nrl in situ probe template amplicon Reverse<br>(adds KpnI digest site for plasmid insertion to pCS2+)<br>GGTACCGACCACCTCGTCTTTGCTGA	This paper	'160711.NCBI.ISHnrl.R
Primer: nr2e3 in situ probe template amplicon Forward<br>CCGAGAGTGTATATGAGACCTCAG	This paper	
Primer: nr2e3 in situ probe template amplicon Reverse (adds T7 promoter)<br>TAATACGACTCACTATAGGGGGGAGGGAAGGAGAAGTAATAGTC	This paper	
Primer: ua3162 genotyping Forward<br>GTCACTTCACAGTTCCCGGT	This paper	20170712.ua3162.F1
Primer: ua3162 genotyping Reverse<br>TCTTCAGCGTCCGTCGTTTC	This paper	20170712.ua3162.R2
Oligonucleotide: nrl-specific gRNA template component1<br>ATTTAGGTGACACTATAGGGGTTCACGCCGAGATGACGTTTTAGAGCTAGAAATAGCAAG	This paper	20160126.nrl.Exon1.1
Oligonucleotide: nrl-specific gRNA template component2<br>ATTTAGGTGACACTATAGGGAGGTCCGTCACTCAGCGGTTTTAGAGCTAGAAATAGCAAG	This paper	20160126.nrl.Exon1.2
Oligonucleotide: nrl-specific gRNA template component3<br>ATTTAGGTGACACTATAGCTGGACGGGAGCCCTTCTGGTTTTAGAGCTAGAAATAGCAAG	This paper	20160126.nrl.Exon1.3
Oligonucleotide: gfp-specific gRNA template component<br>ATTTAGGTGACACTATAGACCAGGATGGGCACCACCCGTTTTAGAGCTAGAAATAGCAAG	This paper	CR.GFP.GA5'.break
Oligonucleotide: first mafbb-specific gRNA template component<br>ATTTAGGTGACACTATAGCTCGGGCTGAAGCTCGGCGGTTTTAGAGCTAGAAATAGCAAG	This paper	CR.mafbb.no25
Oligonucleotide: second mafbb-specific gRNA template component<br>ATTTAGGTGACACTATATATCGTCCTTGCTCATCCCGGTTTTAGAGCTAGAAATAGCAAG	This paper	CR.mafbb.no2

Oligonucleotide: constant gRNA template component AAAAGCACCGACTCGGTGCCACTTTTTCAAGTTGATAACGGACTAGCCTTATTTTAACTTGCTATTTCTAGCTCTAAAAC	Gagnon et al., 2014 (Gagnon et al., 2014)	Constant oligonucleotide
Morpholino: nrl splice-blocking ACGTGTCAGATCATACCTGTGAAGT	This paper	N/A
Primer: to detect nrl splice-blocked transcripts Forward ATGCCACCTCTCTGGAGGAT	This paper	20161110.nrl.Int1.RetF
Primer: to detect nrl splice-blocked transcripts Reverse TCTGACGGCTTGTTCAGGAC	This paper	20161110.nrl.Int1.RetR
Morpholino: standard control morpholino CCT CTT ACC TCA GTT ACA ATT TAT A	Gene Tools, LLC (Philomath, OR)	Standard Control morpholino
Recombinant DNA		
Plasmid: multisite Gateway-compatible middle element ("pME"): zebrafish nrl CDS	This paper	N/A
Plasmid: pME zebrafish codon-optimized cre recombinase	This paper	N/A
Plasmid: pME zebrafish codon-optimized mouse NRL CDS with 3x N-terminal FLAG tag	This paper	N/A
Plasmid: pME zebrafish codon-optimized chicken MAFA CDS with 3x N-terminal FLAG tag	This paper	N/A
Plasmid: pME zebrafish codon-optimized lamprey MAFBA CDS with 3x N-terminal FLAG tag	This paper	N/A
Plasmid: pME zebrafish codon-optimized Drosophila trafficjam CDS with 3x N-terminal FLAG tag	This paper	N/A
Plasmid: pME zebrafish mafk CDS with 3x N-terminal HA tag	This paper	N/A
Plasmid: Tol2kit-style transgenic insert vector: pDestTol2CG2(gnat2:cre.pA)	This paper	N/A
Software and Algorithms		
ZEN 2010 (version 6.0)	Carl Zeiss AG (Oberkochen, Germany)	https://www.zeiss.com/microscopy/int/downloads/zen.html

Gatan DigitalMicrograph, version 1.81.78	Gatan Inc. (Pleasanton, CA)	http://www.gatan.com/products/tem-analysis/gatan-microscopy-suite-software
Geneious R9.1.7	(Kearse et al., 2012)	https://www.geneious.com
Fiji (NIH)	(Schindelin et al., 2012)	http://imagej.net/Fiji
R (version 3.4.1.)	(R Core Team, 2017)	https://www.r-project.org
GraphPad Prism 7.02	GraphPad Software (La Jolla, CA)	https://www.graphpad.com/scientific-software/prism/
Applied Biosystems 7500 Software v1.4.1	Applied Biosystems, Thermofisher	https://www.thermofisher.com/ca/en/home/brands/applied-biosystems.html
Agilent 2100 Bioanalyzer 2100 Expert	Agilent Technologies, (Santa Clara, CA)	https://www.genomics.agilent.com/en/home.jsp

References (Supplemental)

ALLISON, W. T., BARTHEL, L. K., SKEBO, K. M., TAKECHI, M., KAWAMURA, S. & RAYMOND, P. A. 2010. Ontogeny of Cone Photoreceptor Mosaics in Zebrafish. *Journal of Comparative Neurology,* 518**,** 4182-4195.

CONRAD, G. W., BEE, J. A., ROCHE, S. M. & TEILLET, M. A. 1993. Fabrication of microscalpels by electrolysis of tungsten wire in a meniscus. *J Neurosci Methods,* 50**,** 123-7.

DUVAL, M. G., CHUNG, H., LEHMANN, O. J. & ALLISON, W. T. 2013. Longitudinal fluorescent observation of retinal degeneration and regeneration in zebrafish using fundus lens imaging. *Mol Vis,* 19**,** 1082-95.

DUVAL, M. G., GILBERT, M. J., WATSON, D. E., ZERULLA, T. C., TIERNEY, K. B. & ALLISON, W. T. 2014. Growth differentiation factor 6 as a putative risk factor in neuromuscular degeneration. *PLoS One,* 9**,** e89183.

FLEISCH, V. C., LEIGHTON, P. L., WANG, H., PILLAY, L. M., RITZEL, R. G., BHINDER, G., ROY, B., TIERNEY, K. B., ALI, D. W., WASKIEWICZ, A. J. & ALLISON, W. T. 2013. Targeted mutation of the gene encoding prion protein in zebrafish reveals a conserved role in neuron excitability. *Neurobiol Dis,* 55**,** 11-25.

FRASER, B., DUVAL, M. G., WANG, H. & ALLISON, W. T. 2013. Regeneration of cone photoreceptors when cell ablation is primarily restricted to a particular cone subtype. *Plos One,* 8**,** e55410.

GAGNON, J. A., VALEN, E., THYME, S. B., HUANG, P., AKHMETOVA, L., PAULI, A., MONTAGUE, T. G., ZIMMERMAN, S., RICHTER, C. & SCHIER, A. F. 2014. Efficient mutagenesis by Cas9 protein-mediated oligonucleotide insertion and large-scale assessment of single-guide RNAs. *PLoS One,* 9**,** e98186.

KEARSE, M., MOIR, R., WILSON, A., STONES-HAVAS, S., CHEUNG, M., STURROCK, S., BUXTON, S., COOPER, A., MARKOWITZ, S., DURAN, C., THIERER, T., ASHTON, B., MEINTJES, P. & DRUMMOND, A. 2012. Geneious Basic: an integrated and extendable desktop software platform for the organization and analysis of sequence data. *Bioinformatics,* 28**,** 1647-9.

KENNEDY, B. N., ALVAREZ, Y., BROCKERHOFF, S. E., STEARNS, G. W., SAPETTO-REBOW, B., TAYLOR, M. R. & HURLEY, J. B. 2007. Identification of a zebrafish cone photoreceptor-specific promoter and genetic rescue of achromatopsia in the nof mutant. *Invest Ophthalmol Vis Sci,* 48**,** 522-9.

KWAN, K. M., FUJIMOTO, E., GRABHER, C., MANGUM, B. D., HARDY, M. E., CAMPBELL, D. S., PARANT, J. M., YOST, H. J., KANKI, J. P. & CHIEN, C. B. 2007. The Tol2kit: a multisite gateway-based construction kit for Tol2 transposon transgenesis constructs. *Dev Dyn,* 236**,** 3088-99.

PILLAY, L. M., SELLAND, L. G., FLEISCH, V. C., LEIGHTON, P. L., CHENG, C. S., FAMULSKI, J. K., RITZEL, R. G., MARCH, L. D., WANG, H., ALLISON, W. T. & WASKIEWICZ, A. J. 2013. Evaluating the Mutagenic Activity of Targeted Endonucleases Containing a Sharkey FokI Cleavage Domain Variant in Zebrafish. *Zebrafish,* 10**,** 353-64.

RAYMOND, P. A., BARTHEL, L. K., ROUNSIFER, M. E., SULLIVAN, S. A. & KNIGHT, J. K. 1993. Expression of rod and cone visual pigments in goldfish and zebrafish: a rhodopsin-like gene is expressed in cones. *Neuron,* 10**,** 1161-74.

SUZUKI, S. C., BLECKERT, A., WILLIAMS, P. R., TAKECHI, M., KAWAMURA, S. & WONG, R. O. 2013. Cone photoreceptor types in zebrafish are generated by symmetric terminal divisions of dedicated precursors. *Proc Natl Acad Sci U S A,* 110**,** 15109-14.
